# Signalling crosstalk during early tumorigenesis in the absence of Polycomb silencing

**DOI:** 10.1371/journal.pgen.1007187

**Published:** 2018-01-22

**Authors:** Jorge V. Beira, Joana Torres, Renato Paro

**Affiliations:** 1 ETH Zürich, Department of Biosystems Science and Engineering, MattenstrasseBasel, Switzerland; 2 Faculty of Science, University of Basel, KlingelbergstrasseBasel, Switzerland; New York University, UNITED STATES

## Abstract

In response to stress and injury a coordinated activation of conserved signalling modules, such as JNK and JAK/STAT, is critical to trigger regenerative tissue restoration. While these pathways rebuild homeostasis and promote faithful organ recovery, it is intriguing that they also become activated in various tumour conditions. Therefore, it is crucial to understand how similar pathways can achieve context-dependent functional outputs, likely depending on cellular states. Compromised chromatin regulation, upon removal of the Polycomb group member *polyhomeotic*, leads to tumour formation with ectopic activation of JNK signalling, mediated by *egr*/*grnd*, in addition to JAK/STAT and Notch. Employing quantitative analyses, we show that blocking ectopic signalling impairs *ph* tumour growth. Furthermore, JAK/STAT functions in parallel to JNK, while Notch relies on JNK. Here, we reveal a signalling hierarchy in *ph* tumours that is distinct from the regenerative processes regulated by these pathways. Absence of *ph* renders a permissive state for expression of target genes, but our results suggest that both loss of repression and the presence of activators may collectively regulate gene expression during tumorigenesis. Further dissecting the effect of signalling, developmental or stress-induced factors will thus elucidate the regulation of physiological responses and the contribution of context-specific cellular states.

## Author summary

Signalling pathways modulate cellular responses in developmental and regenerative processes but can also be misused by cancer cells to promote tissue invasion and growth. How signalling outputs are integrated to direct a variety of responses ranging from organ repair to tumour onset remains elusive. Loss of the epigenetic Polycomb repressive complex PRC1, which regulates cell identity during normal development, leads to tumour formation in epithelial tissues, supported by aberrant signalling activities. Employing quantitative and genetic methods, we dissect the signalling hierarchy between the pathways involved in tumour growth, namely JNK, JAK/STAT and Notch. We find that JNK activation in *ph* tumours is mediated by *eiger* and its receptor *grindelwald*. We identify a signalling hierarchy where JNK and JAK/STAT function in parallel in the tumour model, which is in contrast to a coordinated sequential activation of the same pathways in a regenerative context. Our data also reveal a higher regulatory complexity underlying tumour growth, as signalling-dependent activators can contribute to tumorigenic gene expression in addition to loss of repression. This dual mode of regulation may be relevant in a range of tumour models and organisms, since aberrant signalling and deregulated gene expression programs are general hallmarks of cancer tissues.

## Introduction

Tissues are capable of restoring homeostasis after damage or stress through coordinated signalling responses. However, cells sometimes deviate from this normal repair path to attain a tumorigenic state, often entailing the usage of similar signalling modules. Cancer cells become self-sufficient in controlling cell division and metabolic programs, hijacking growth pathways and evading cell death. How cells acquire unconventional mechanisms during transformation to direct tumour-sustaining signals is still largely unknown.

*Drosophila* tumour models revealed key oncogenic features in tissues, which appear conserved across species. These include loss of cell polarity, compromised epithelial architecture and oncogenic cooperation. For instance, activation of known tumour drivers such as Ras, Notch, Myc or EGFR lead to benign hyperplasia in imaginal discs, but in combination with loss of epithelial integrity result in neoplastic growth [1–4]. Epithelial tumours in humans also lose polarity as they acquire malignant properties, pointing towards the importance of altered cellular interactions for disease progression in tissues harbouring pre-cancerous lesions. The interactions between defective cells and the environment as well as the adjacent healthy tissue have been increasingly recognized to be relevant for tumour development [5–8]. *Drosophila* tissues are particularly well suited to address the events that occur during the early steps of tumorigenesis, which are difficult to dissect in human cancers that are often detected only at much later stages.

A number of ectopic signalling activities have been reported in several tumour models, with some shared morphological and molecular similarities. Cell overproliferation and defects in epithelial architecture are observed in tissues lacking functional polarity components (such as *scribble*, *dlg* or *lgl*) in combination with activated forms of oncogenes like *ras*^*V12*^ or *N*^*act*^ [3,9–11]. Invasive properties of polarity-deficient cells have been shown to depend on JNK signalling [12,13]. Tumour cell clusters sort out from the neighbouring wild-type tissue, as shown by clonal analysis, and display rounded shapes that resemble cysts [14,15]. Interestingly, mutations in epigenetic silencers, including Polycomb group (PcG) members, also lead to tumour formation in epithelial tissues, without the need for additional oncogenic cooperation [16,17]. These tumours share many of the previously mentioned features, as clones displaying polarity defects grow beyond the normal boundaries of the host tissue leading to its deformation. Activation of JAK/STAT and Notch signalling has been reported in tumours lacking a functional Polycomb Repressive Complex 1 (PRC1) due to loss of *polyhomeotic* (*ph*), which contribute to their growth [16–18]. Other signalling pathways, including EGFR, Ras, Hippo and Myc have been previously examined, but were reported to be unchanged or only having some ectopic activation in *ph* tumours [17]. It becomes clear that faithful chromatin regulation is key during development and tissue homeostasis, pointing towards the consequences of abnormal epigenetic control in compromised tissue function or rendering tumour formation.

Despite the recurrent observations of ectopic signalling activities in tumours, their contribution during early tumorigenesis has rarely been addressed. It is puzzling how developmental signalling ensures the formation of normal tissue structures but lead to abnormal growth when activated by tumour cells. For example, regeneration of imaginal discs in *Drosophila* requires JNK signalling, which in turn activates the JAK/STAT pathway [19]. Both pathways regulate the regenerative response, after transiently overcoming Polycomb silencing, and allow cell fate reallocation. However, they also become activated in tumours resulting from permanent abrogation of Polycomb function. Here, we sought to functionally address how ectopic JNK, JAK/STAT and Notch signalling contribute to *ph* tumour growth and which relationships exist among these pathways. We can thus identify signalling hierarchies that are context-specific and determine the necessary elements required for tumour development that differ from regeneration. Our results support a dual relevance for ectopic signalling pathways and the loss of the PRC1 silencing in promoting gene expression programs supporting tumorigenesis.

## Results

### In the absence of the PRC1 member *ph*, tumours display ectopic JNK, JAK/STAT and Notch signalling

**Fig 1:**
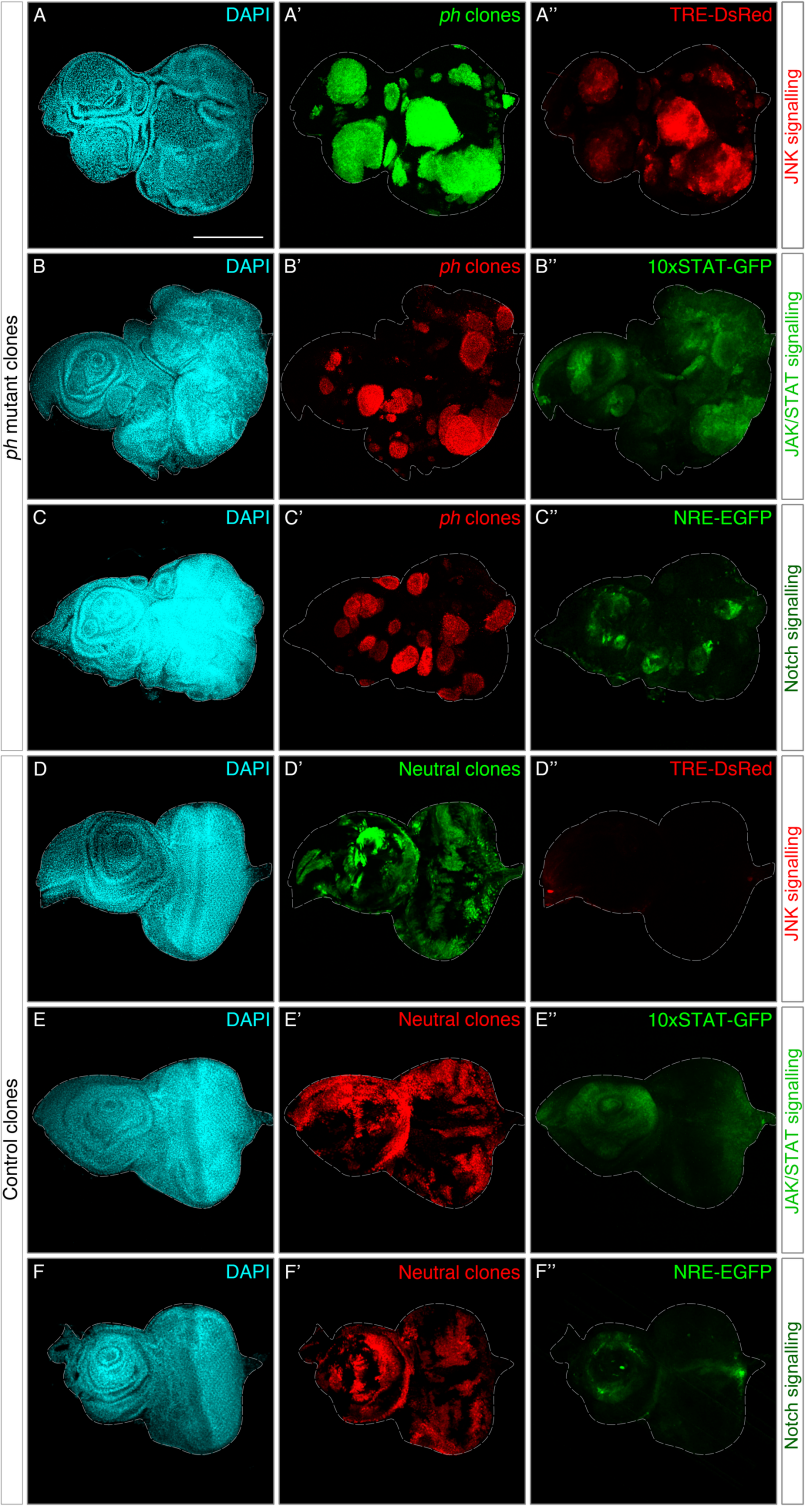
Reporters of JNK, JAK/STAT and Notch signalling are ectopically activated in *ph* mutant clones. **(A-C)** Eye-antennal discs where *ph* mutant clones (MARCM) were generated leading to tumour formation. **(D-F)** Control discs with similarly induced neutral clones. Tissue morphology is shown in the left panels (DAPI). Marked clones are depicted in the central panels, either with act>>GFP (A’/D’) or act>>RFP depending on the fluorescence of each signalling reporter, shown on the right panels. **(A”,D”):** Expression pattern of a JNK reporter (TRE-DsRed) in discs with *ph* mutant (A) or control (D) clones. **(B”,E”):** Pattern of the 10xSTAT-GFP reporter in discs with *ph* mutant (B) or neutral (E) clones. **(C”,F”):** Notch signalling as measured with a NRE-GFP reporter in eye discs carrying *ph* mutant (C) or control clones (F). Scale bar represents 200 μm.

Loss of the PRC1 component *Polyhomeotic* (*ph*) leads to tumour formation characterized by compromised epithelial integrity and cell polarity, neoplastic overgrowth and poor differentiation capacity [16,17]. To monitor ectopic signalling activities, we combined fluorescent reporters with an established *ph* tumour model by generating MARCM clones in the developing epithelium of eye imaginal discs [20]. Clones mutant for *ph* form rounded structures that sort out from the adjacent tissue monolayer, forming a multi-layered tissue with defective epithelial architecture that can be visualized with a nuclear DAPI staining (Fig 1A–1C). This is in contrast to the organized tissue architecture of eye-antennal discs with neutral clones (control clones that only express GFP) (Fig 1D–1F). Tumour-bearing discs display severe abnormalities, as *ph* clones grow beyond the boundaries that normally define the characteristic shape of eye-antennal discs (Fig 1A’–1C’). By contrast, neutral clones respect the epithelial plane and display a patchy appearance (Fig 1D’–1F’).

JNK signalling has been widely reported to become activated in response to tissue damage, associated with loss of epithelial integrity and often in tumours [9,12]. We monitored JNK signalling with a fluorescent reporter that is driven by the transcription factor AP-1, the canonical heterodimer activated by the JNK pathway [21]. Ectopic expression of the JNK reporter was largely detected to overlap with *ph* clones (Fig 1A’ and 1A”), while the reporter was virtually silent in wild-type eye discs and unaffected by neutral clones (Fig 1D’ and 1D”).

To determine how JNK becomes activated upon loss of *ph*, we considered a possible role for *eiger* and its receptor *grindelwald*, which have been shown to mediate JNK activation in other tissue damage contexts [22]. Indeed, we found that this ligand-receptor pair is also required for JNK activation in *ph* clones, since the JNK reporter is downregulated when knocking-down either gene in *ph* tumours (S1E and S1F Fig). Furthermore, as Egr-dependent expression of Matrix metalloprotease-1 (Mmp1) has been linked with invasiveness in *ras*^*V12*^/*scrib* tumours [13,22], we assessed whether this target would also require *egr*/*grnd*-mediated JNK activation in *ph* tumours. Mmp1 staining revealed that its upregulation in *ph* tumours (S1A Fig) is prevented by blocking JNK signaling either with *bsk*^*DN*^ (S1B Fig) or upon knocking down *egr* or *grnd* (S1C and S1D Fig). These data show that functional JNK activation depends on *eiger*/*grindelwald*, and is required for Mmp1 upregulation.

We confirmed that JAK/STAT and Notch signalling were activated in eye discs with *ph* clones, using available reporters for each pathway [23,24]. The STAT reporter was expressed in most *ph* clones but also extended beyond them in some instances (Fig 1B’ and 1B”), in line with a wider range of activation due to secretion of *upd* ligands [17]. The reporter is normally detected in a specific pattern in wild-type discs, namely in the antenna region and some photoreceptors (Fig 1E”), which is clearly altered in discs with *ph* clones (Fig 1B”). Finally, the Notch reporter was detected in a subset of *ph* clones (Fig 1C’ and 1C”), while its endogenous expression was mostly restricted to a circle prefiguring the developing antennal region (Fig 1F’ and 1F”). The ectopic activation of JNK, JAK/STAT and Notch signalling in *ph* clones prompted us to ask whether each pathway is important for tumour development or if some are rather peripheral to tumorigenesis.

### Impaired JNK, JAK/STAT or Notch signalling impact on *ph* tumour growth

**Fig 2:**
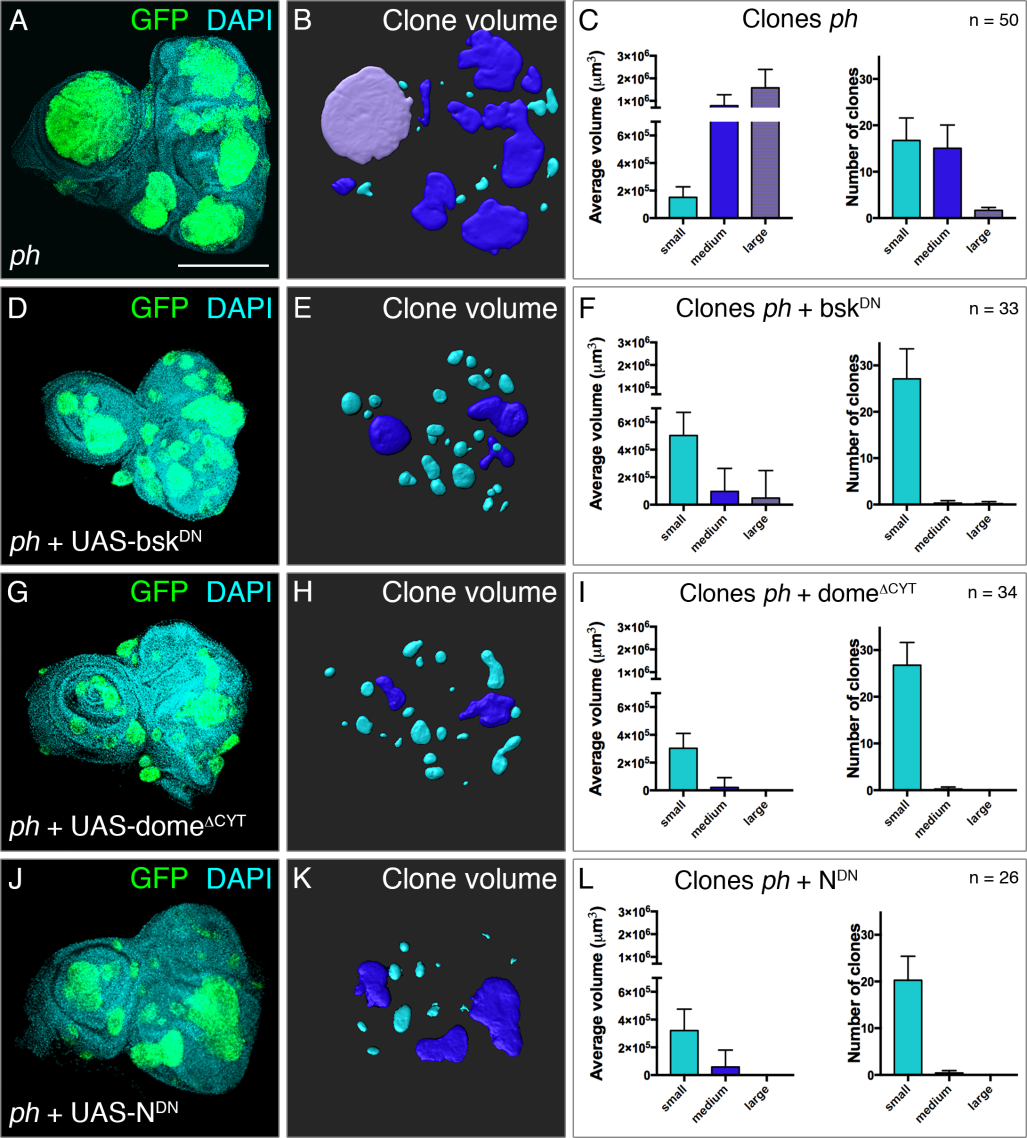
Tumour growth is compromised upon blocking signal transduction of JNK, JAK/STAT or Notch pathways. **(A-B)** A large number of eye discs with *ph* clones was used to obtain quantitative measurements of clone volume and number. (A) Disc morphology becomes apparent with DAPI staining, mutant clones are marked by GFP expression. (B) Clone volumes were pseudo-coloured corresponding to three classes reflecting total volume: small (cyan), medium (dark blue) and large (purple). **(C)** Distribution of average volumes (left) taken by clones in each of the three categories per disc, and average number of clones in each category per disc (right). The bar plots for each category follow the same colour scheme for the clones depicted in the central panels. Similar analyses were performed upon blocking JNK, JAK/STAT or Notch signalling. The corresponding discs, clone volumes and measurements are shown for discs with *ph* clones expressing UAS-bsk^DN^ **(D-F)**, UAS-dome^ΔCYT^ **(G-I)** or UAS-N^DN^ **(J-L)**; error bars represent standard deviations. Scale bar represents 200 μm.

To functionally test the contribution of each pathway to *ph* tumour growth, we used genetic tools to block signal transduction in *ph* mutant clones and examined their effect. We used the established *ph* tumour model in eye-antennal discs by inducing eyFlp-driven MARCM *ph*^*505*^ null clones surrounded by normal tissue, and also allowing restricted expression of additional transgenes within the mutant clones [16,17]. Due to the neoplastic nature of *ph* clones, which spatially grow beyond the epithelial plane, we implemented a quantitative framework to measure clone volume in a large number of discs. While clonal area is often the main parameter used to measure growth differences, the aberrant tumoral growth beyond the epithelial plane prompted us to recognise the need for volume measurements in this context. This approach takes into account the intrinsic variability of tumour tissues and provides a robust ground with statistic power to measure differences in tumour growth upon genetic abrogation of signalling modules (Fig 2 and S2 Fig).

First, we devised a standard method to capture the 3D character of *ph* clones and measure their number and respective volume across 50 *ph*-containing eye discs (see Materials and Methods) (Fig 2A and S2A and S2B Fig). We observed a range of different clone sizes in each disc, reflecting the intrinsic diversity observed previously. We identified three categories that describe the observed distribution in clone volumes (small, medium and large), which were pseudo-coloured according to their dimensions (Fig 2B). On average, there were around 26–33 clones per disc (distributed among the three classes), with only one or two large clones consistently found in each disc (S2B and S2B’ Fig), but which made up a significant proportion of the total tumoral tissue (up to 45% of the total GFP volume per disc) (Fig 2C and S2B Fig). The distribution of tumour volume into three categories was generally consistent (S2A and S2B Fig), so even though the total volume might vary across discs, the proportional contribution remained (S2B’ Fig).

We then extended this quantitative framework to measure the consequences of blocking each pathway to tumour growth. JNK signalling was blocked by expressing a dominant negative form of the *basket* kinase (UAS-bsk^DN^) [25]. We observed a decrease in the average clone volume (Fig 2D and pseudo-coloured volumes in Fig 2E), although the total number of clones per disc remained generally constant. We observed a decrease in the volume taken up by medium and large *ph* clones upon blocking JNK (Fig 2F), while the tumour volume made up by small clones increased in comparison to the baseline *ph* condition (without block) (Fig 2C). In addition, this is also reflected by a decrease in the number of medium and large clones (compare Fig 2F–2C), which were rarely found, and a corresponding increase in the number of small clones. Thus, upon blocking JNK signalling, there was a volume shift from medium and large categories to clones with smaller volume. As we established that JNK activation is dependent on the TNF receptor *grnd* and its ligand *egr*, we also measured the effect on *ph* tumour volume upon knockdown. We confirmed their impact on tumour growth (S2H–S2K Fig), which is also clearly observed in comparison to blocking the three pathways (tumour volume measurements summarized in S2L Fig).

JAK/STAT signalling was perturbed through expression of a mutated form of the sole *Drosophila* receptor, *domeless* (UAS-dome^ΔCYT^) [26], as a way to block the pathway irrespective of one or more ligands that could activate it. As with JNK, we similarly found that blocking JAK/STAT signalling resulted in a reduction of clone volume, as we found mostly small and a few medium clones but not large ones (Fig 2G–2I). The effect was more pronounced with impaired JAK/STAT, depicted for example by an even smaller volume taken up by medium clones in this condition (compare Fig 2I to 2C). As for the Notch pathway, we expressed a dominant negative transgene of the receptor in *ph* clones (UAS-N^DN^) [27], which also yielded clones to mostly shift to the small volume category (Fig 2J–2L). We also noted that, in this case, the number of clones was slightly reduced in comparison with blocking the other two pathways, even though a similar trend to detect mostly clones with small volume was observed. As controls, we quantified the size of neutral clones expressing either of the constructs used to block the three pathways. We verified that these clones showed normal morphology, stayed in the epithelial plane, and that the area taken by neutral clones in eye discs did not significantly change upon blocking these pathways (S2C–S2G Fig).

These data show that all three pathways activated in *ph* clones (JNK, JAK/STAT and N) contribute to tumour growth, as impairing signalling through any of the three cascades impacts on clone volume. Although medium and large clones are commonly seen in *ph* clones where these pathways are active (Fig 2C), there is a shift towards smaller clones upon impaired signalling (compare Fig 2F, 2I, 2L to 2C), suggesting that clones are not able to grow as much in the absence of these signalling modules. As all three pathways support *ph* tumour development, we took advantage of this simple model to determine what relationships might exist among them and thus define context-specific hierarchies relevant to tumorigenesis.

### JNK signalling acts in parallel to JAK/STAT but upstream of Notch

**Fig 3:**
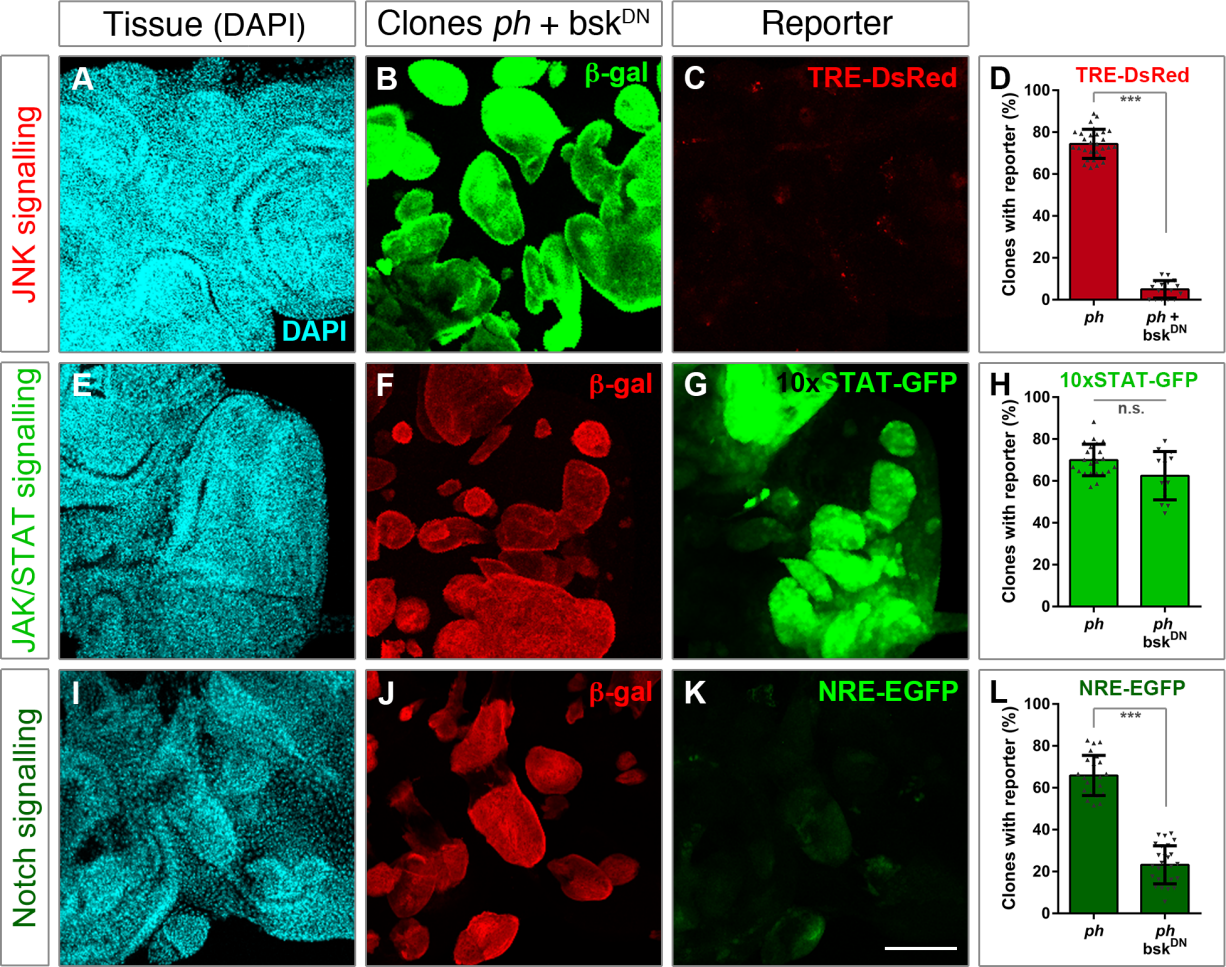
Blocking JNK signalling does not impair JAK/STAT but impacts on Notch. The effect of blocking JNK signalling was assessed using reporters for the three pathways. **(A-D)** TRE-DsRed was used for JNK signalling, **(E-H)** the 10xSTAT-GFP reporter for JAK/STAT, and **(I-L)** NRE-EGFP as a read-out of the Notch pathway. The left panels show tissue morphology with DAPI staining (A,E,I), followed by panels depicting the distribution of *ph* clones expressing bsk^DN^. Clones are marked by β-gal expression, either in green (B) or red (F,J) depending on the fluorescence of the corresponding reporter. For each condition, the percentage of *ph* clones showing reporter activity was measured, as shown in comparison to *ph* clones alone (right panels). The bar plots represent the average proportion of clones with reporter expression; error bars represent standard deviations; *** p<0.005 (Student t-test). Scale bar represents 100 μm.

To dissect whether these three functionally relevant pathways act independently or show levels of crosstalk, we combined specific signalling read-outs with a blockage of each cascade. We started by testing the effect of blocking JNK in the other two modules, by expressing UAS-bsk^DN^ in *ph* clones. We established how broadly activated was each signalling reporter in tumours by determining the proportion of *ph* clones showing reporter activity in respect to all marked clones across several discs. We could then measure the effect on each signalling reporter upon blocking individual pathways. The JNK pathway was widely abrogated, as the TRE-DsRed reporter was robustly downregulated in *ph* clones expressing UAS-bsk^DN^ (compare Figs 3A–3D to 1A–1A”).

Next, we examined how blocking JNK impacted on the other two cascades. Indeed, JNK has been shown to act upstream of JAK/STAT in response to tissue damage and promote regeneration by upregulating the *unpaired* ligand [19]. However, the 10xSTAT-GFP reporter was comparably detected upon expression of bsk^DN^ as in *ph* clones alone (Fig 3E–3H). This suggested that the same signalling pathways that are subjected to different hierarchical controls specify either regenerative or tumorigenic development.

Finally, we observed a reduced expression of the Notch reporter (NRE-EGFP) when co-expressing bsk^DN^ in *ph* clones (Fig 3I–3L). These data suggested that, although JAK/STAT functions in parallel to JNK in *ph* tumours, the Notch pathway relies on JNK signalling and thus may function downstream.

To further explore the connection between JNK and Notch, we asked whether this could be ligand-dependent or independent. As previously shown [16], we could also verify that Serrate is upregulated in *ph* tumours (S3D–S3F Fig), while Delta expression is similar to that of control discs (S3A–S3C Fig). Thus, we asked if this could depend on JNK. We first hypothesized it could indeed act through Serrate transcriptional regulation, but we did not observe a marked effect on Ser immunoreactivity when blocking JNK (S3D–S3F Fig), apart from clones getting smaller. This pointed to an alternative target, so we tested the receptor itself, using an antibody against the Notch intra-cellular domain (NICD). Discs with *ph* clones showed ectopic NICD signal (S3H Fig), and we observed a reduction NICD signal in *ph* clones upon blocking JNK (S3I and S3J Fig). The pattern was more comparable to that of control discs with neutral clones (S3G Fig), although we noted that some variability, namely in bigger clones. These data suggest that JNK can exert an effect at the level of NICD regulation, although we cannot exclude indirect regulation since several other factors are involved in its processing. Nevertheless, it points to ligand-independent regulation, as Delta and Serrate were vastly unaffected when blocking JNK signalling.

### JAK/STAT signalling functions independently of JNK and Notch

**Fig 4:**
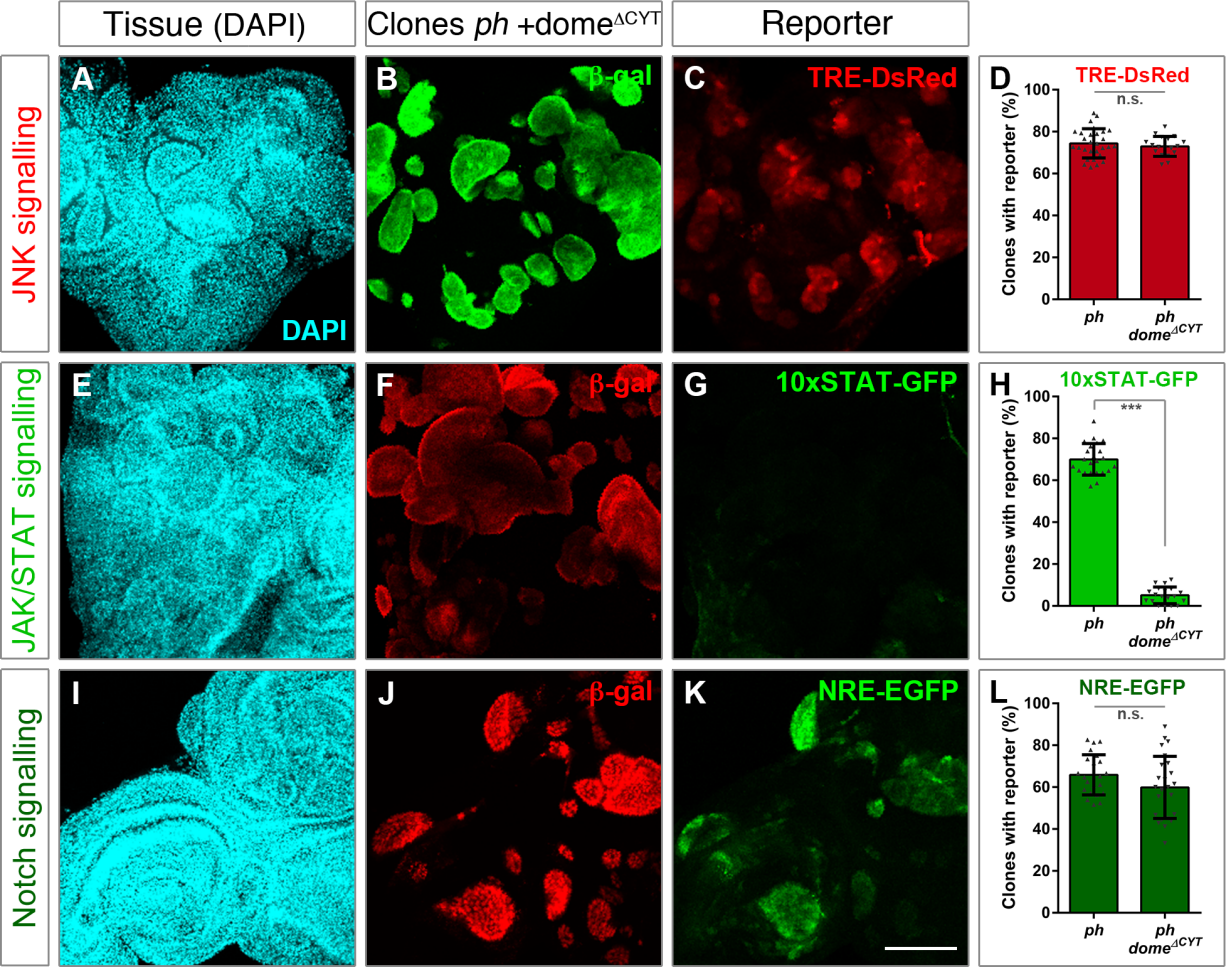
JAK/STAT signalling functions in parallel to the JNK and Notch pathways. Expression of UAS-dome^ΔCYT^ was used to block JAK/STAT signalling in *ph* clones. The effects were examined using reporters for the three pathways, with TRE-DsRed for JNK **(A-D)**, 10xSTAT-GFP for JAK/STAT **(E-H)** and NRE-EGFP for Notch signalling **(I-L)**. DAPI staining is shown in the left panels to highlight disc morphology (A,E,I). Clones lacking *ph* and dome^ΔCYT^ are marked by β-gal in green (B) or red (F,J) depending on the reporters’ fluorescence. The percentage of *ph* clones with JAK/STAT signalling is reduced (H) but the reporters for JNK and N are similarly detected upon blocking JAK/STAT. Bar plots represent the average percentage of clones with reporter expression either in *ph* clones alone or with dome^ΔCYT^ expression; error bars represent standard deviations; *** p<0.005 (Student t-test). Scale bar represents 100 μm.

We blocked JAK/STAT signalling by expressing the previously mentioned dominant negative receptor transgene (UAS-dome^ΔCYT^), and examined its consequence with reporters of all three pathways. As a reciprocal test to the previous observation that JAK/STAT was little affected by JNK, we now checked the JNK reporter upon expression of dome^ΔCYT^ in *ph* clones. Consistently, the JNK reporter remained active as in *ph* clones alone (Fig 4A–4D), suggesting that JAK/STAT and JNK act independently in *ph* tumours. We also confirmed that expression of dome^ΔCYT^ efficiently abrogated the JAK/STAT reporter in *ph* clones (Fig 4E–4H). We did not observe an effect on the distribution of the Notch reporter in this condition, however (Fig 4I–4L).

These observations strengthen the hypothesis that JAK/STAT signalling functions independently of JNK and Notch in promoting tumorigenesis upon loss of *ph*.

### Impairment of Notch signalling has little effect on JNK and JAK/STAT

**Fig 5:**
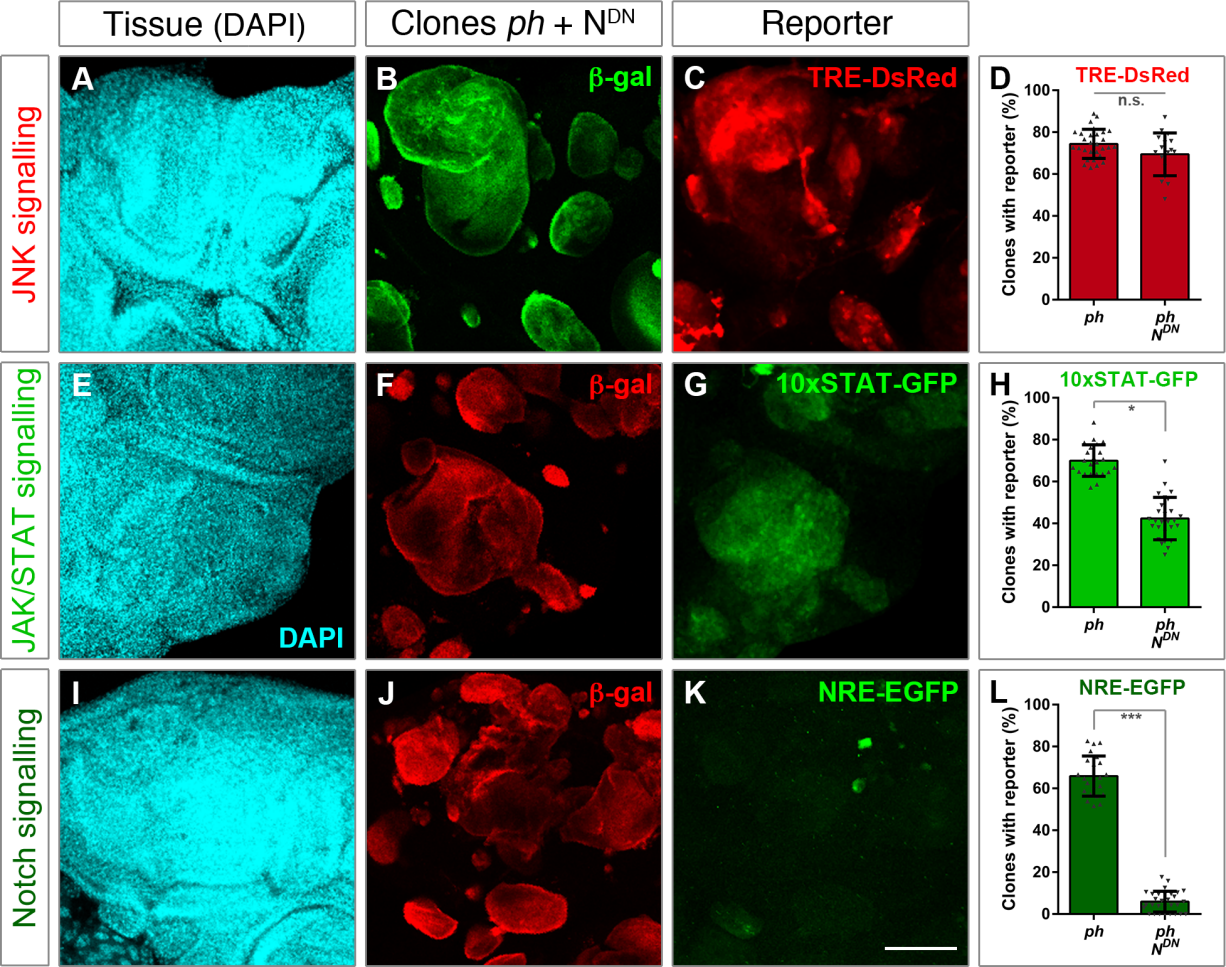
Impairment of Notch signalling has little effect on JNK and JAK/STAT. Upon impaired signal transduction of the Notch pathway, by expressing UAS-N^DN^ in *ph* clones, the effects were assessed using reporters for JNK signalling (TRE-DsRed) **(A-D)** the JAK/STAT pathway (10xSTAT-GFP) **(E-H)** and NRE-EGFP for Notch **(I-L)**. The proportion of clones with expression of the Notch reporter was markedly reduced in comparison to *ph* clones alone (L), serving as a positive control. The JNK reporter was similarly expressed in both conditions (D), and a mild reduction of the JAK/STAT reporter (G) was observed. DAPI staining is shown to depict tissue morphology (left panels), mutant clones are marked by β-gal expression in green (B) or red (F,J) with the remaining channel for respective signalling reporters (C,G,K). The average percentage of clones showing reporter expression was measured both in *ph* clones alone and upon expression of N^DN^, as shown in the bar plots (D,H,L). The error bars represent standard deviation; *** p<0.005, * p<0.01 (Student t-test). Scale bar represents 100 μm.

To examine how Notch signalling could influence the other pathways, we carried out similarly designed experiments using UAS-N^DN^ in *ph* clones. The JNK reporter was detected in a similar proportion of mutant clones as in *ph* tumours alone (Fig 5A–5D). The JAK/STAT reporter was modestly reduced upon blocking Notch signalling in comparison to *ph* clones alone (Fig 5E–5H). However, we noticed that this is likely contributed by downregulation of the reporter mostly in smaller clones, as larger ones still express NRE-EGFP. A clear difference was observed in the NRE-EGFP reporter when expressing N^DN^, confirming signalling blockage for the target pathway (Fig 5I–5L). Thus, blocking Notch does not impinge on JNK and has a modest effect on JAK/STAT signalling reporters. The latter could be due to Notch-dependent upregulation of the *upd* ligand, which could be partly contributing as an additional relay, as was previously established in the eye disc [28–30].

Taken together, the previous set of observations highlight a signalling hierarchy specific to *ph* tumours, where JAK/STAT signalling functions simultaneously but independently to the JNK pathway, unlike previous observations in a regenerating tissue [19]. Our data also suggest that Notch acts downstream of JNK in this context, thus pointing to the possibility that simultaneous JNK and JAK/STAT signalling could lead to a distinct outcome in *ph* tumorigenesis from the previously described sequential function during regeneration.

### Co-activation of JNK and JAK/STAT is not sufficient to trigger tumour phenotypes

To further explore the role of JNK and JAK/STAT signalling in directing cellular responses to damage, we asked whether simultaneous function of these two pathways could be sufficient to drive any tumour-resembling characteristics in epithelial tissues. We were motivated by the differences in outcome, from a JNK-dependent input on the JAK/STAT in tissue regeneration, contrasted by the current observations of simultaneous function of these pathways in *ph* tumours. We thus took a simple approach to test the effect of triggering both pathways simultaneously, attempting to induce a signalling context similar to that of *ph* clones. We focused on JNK and JAK/STAT as these were activated in parallel in tumours, while Notch seemed to have inputs from JNK, and Notch expression leads to tissue hyperplasia on its own, which could lead to confounding effects [10,31,32].

We used the same system as for *ph* clones to facilitate comparison, by generating MARCM eyFlp clones in eye-antennal discs but without the *ph* mutation. Discs with neutral clones (GFP-marked cells) show a normal appearance with a typical planar-organized layer of epithelial cells (S4A–S4C Fig). When activating JNK in such clones, by expressing an activated form of *hemipterous* (UAS-hep^act^) that acts upstream of the main JNK kinase [33], a reduced number of clones was recovered (S4D–S4G Fig), in line with the well-described pro-apoptotic function of JNK that can lead to elimination of cells from the tissue [9,19,34]. We also confirmed that the JNK reporter was detected in or around clones with overactive JNK (S4G Fig).

To trigger JAK/STAT, we induced neutral clones overexpressing the sole JAK kinase *hopscotch* (UAS-hop) [35]. The STAT reporter was detected in these clones, in addition to the endogenous pattern in the eye-antennal disc ([23] and S4J Fig), but the clones remained in the epithelial layer and had patchy shapes similar to neutral clones without additional transgene expression (S4H–S4K Fig).

We then co-expressed UAS-hep^act^ and UAS-hop in otherwise wild-type cells, to over-activate both pathways as we observe in *ph* mutant clones. Even in this condition, discs appeared to broadly retain a normal morphology, and clonal shape was rather similar to that of neutral clones, growing in non-preferential directions within the epithelial plane (S4L–S4N Fig). We did not observe formation of cyst-like structures or local alterations to tissue morphology that could be attributed to the nature of clones in respect to the surrounding wild-type tissue. We noted that more clones were recovered when co-expressing both UAS-hep^act^ and UAS-hop than with UAS-hep^act^ alone, likely due to increased survival of cells with JNK signalling conferred by JAK/STAT, as recently reported in response to damage [36]. Nevertheless, we could not identify characteristic tumour features in tissues carrying clones with simultaneous expression of JNK and JAK/STAT components.

These data suggest that co-induction of JNK and JAK/STAT signalling is not sufficient by itself to promote tumour-associated malformations, even though both pathways are ectopically activated in *ph* tumours. Therefore, epithelial tumour initiation likely depends on additional elements that are not fully reunited by collective function of these pathways. The chromatin state is a likely determining factor to modulate signalling responses, which is clearly altered in clones lacking *ph*. In light of these considerations, we sought to identify additional requirements to JNK and JAK/STAT in the development of *ph* tumours.

### Loss of PRC1 is not sufficient for generalized de-repression of PcG targets

Alterations in gene expression programs are intrinsic to tumorigenesis, and abrogation of PRC1 function in *ph* clones will expectedly lead to chromatin changes that support tumour initiation. As Polycomb complexes target hundreds of developmental and signalling genes, a general assumption in the field has laid the expectation that PRC1 targets would become de-repressed in its absence [16,17,37]. Following the lead that there must be additional requirements for *ph* tumour formation than concomitant JNK and JAK/STAT, we revisited the state of known Polycomb targets in *ph* clones. An early report of *ph* clones in wing discs showed a compartment-specific upregulation of established target genes, including *engrailed*, *hedgehog* (*hh*), *patched* and *decapentaplegic* (*dpp*) [15]. This suggested that a more complex modulation, potentially dependent on additional activators, could underlie the re-expression of Polycomb targets in distinct tissues or contexts.

**Fig 6:**
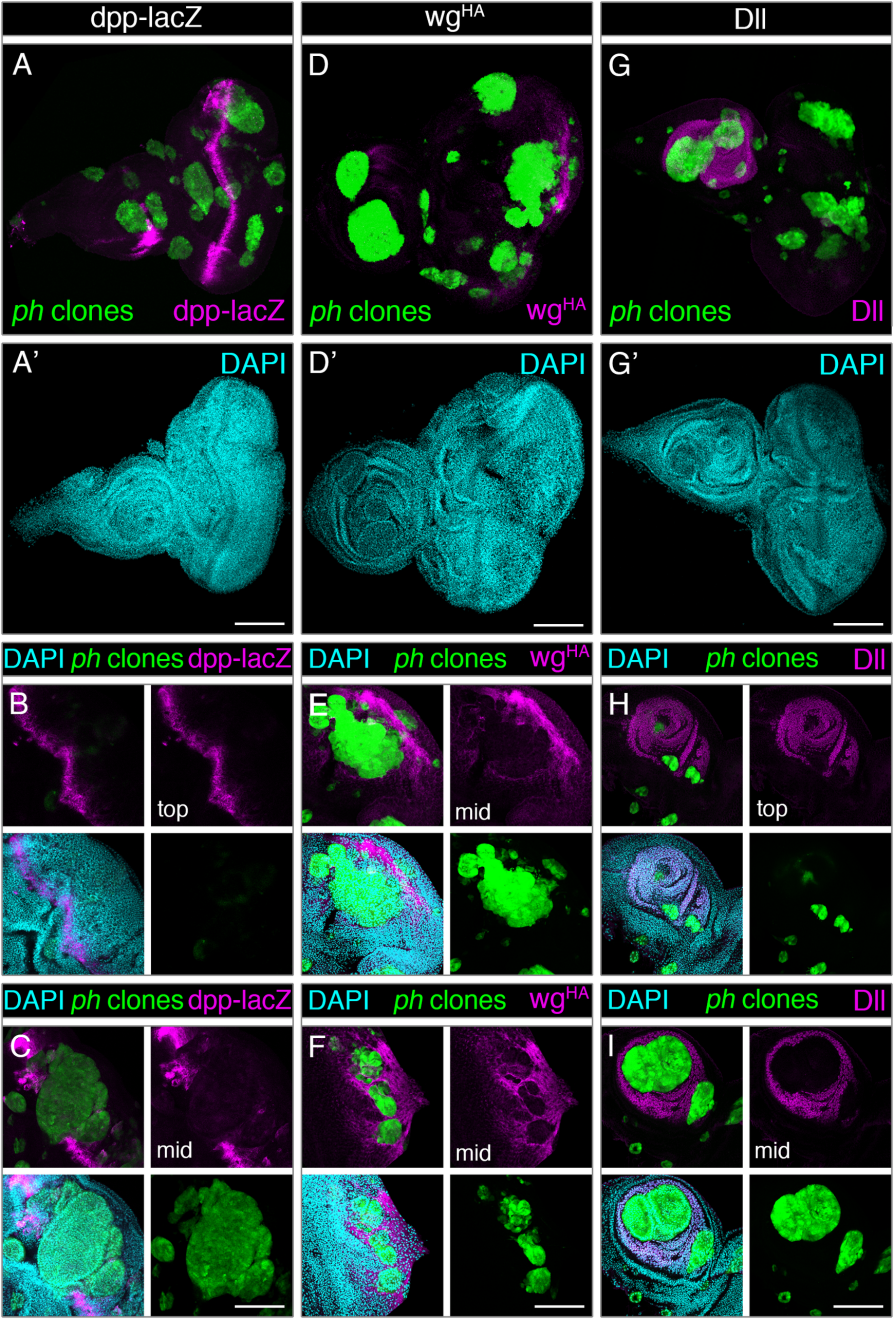
Loss of PRC1 is not sufficient for generalized de-repression of PcG target genes. Expression of three known PcG targets was examined in discs carrying *ph* mutant clones, namely *dpp* (left panels), *wingless* (central panels) and *Distal-less* (right panels). **(A-A’)** Expression of the dpp-lacZ reporter (magenta) was restricted to stereotypical regions of wild-type discs near the morphogenetic furrow and a section of the antenna, despite the broad distribution of *ph* clones (GFP, green) in the disc. DAPI staining is shown in cyan. **(B-C)** Close-up sections of the same eye disc show that the dpp-lacZ reporter (magenta) was observed in sections above the GFP-marked *ph* clone (green) (top) (B), but in a middle section (C) where the cells lacking *ph* are visible (green) the expression of dpp-lacZ is interrupted and only detected in the surrounding wild-type cells. **(D-D’)** Staining for endogenously HA-tagged Wg (magenta) was not observed in the majority of *ph* clones (green). **(E-F)** close-up sections of eye discs show wg^HA^ signal in a characteristic posterior region of the eye where photoreceptors normally differentiate, and reduced signal is detected in *ph* mutant cells arising in close proximity or within these regions. **(G-G’)** Dll staining (magenta) is restricted to the antenna region even in eye discs with *ph* clones (green), which did not show ectopic Dll signal. **(H,I)** The stereotypical Dll signal in the antenna is clear in a top section of a disc (H), but closer to the middle of the same disc (I) the growth of *ph* clones (green) in this region led to interruption of Dll staining in *ph* mutant cells. Scale bars represent 200 μm.

We analysed the expression of known PcG targets in eye-antennal discs with *ph* clones. *dpp* and *wingless* (*wg*) are PcG targets that encode ligands for conserved pathways (homologs of TGF-β and Wnt signalling) involved in regulating growth and patterning of imaginal discs [8]. We combined an available *dpp* reporter, dpp-lacZ, in a *ph* mutant background and checked its expression in eye discs with *ph* clones. The *dpp* reporter is normally expressed in a section of the antennal region as well as the morphogenetic furrow of the eye (S5A and S5B Fig), which we also observed in discs with *ph* clones (Fig 6A and 6A’). However, dpp-lacZ was not detected in the vast majority of *ph* clones, and there were instances where the typical row of expression along the furrow seemed to be deformed by the neighbouring mutant clones which seemed to push adjacent cells away from their usual pattern (Fig 6A). We examined the tissues in further detail to uncover the 3D structures formed by *ph* tumours, by analysing z-sections closer to the disc surface (‘top’) or towards the middle (‘mid’). The *dpp* reporter was expressed in a row of cells at the surface where no clone was yet present (Fig 6B), but the expression of dpp-lacZ was interrupted by a GFP-marked *ph* clone at a deeper z-position in the same disc (Fig 6C). This suggested that *ph* clones did not globally express dpp-lacZ throughout the eye discs.

To assess *wg* expression in *ph* clones, we resorted to a viable genetically engineered fly strain with an endogenously tagged *wg* version, *wg*^HA^ [38]. The characteristic *wg* expression pattern in posterior photoreceptors and in a defined location of the antenna was similarly confirmed, both in wild-type discs as in discs with neutral clones (S5C and S5D Fig). However, *ph* clones generally lacked *wg*^*HA*^ staining (Fig 6D and 6D’). By taking a closer inspection of tissue sections, we observed that the expression of *wg*^HA^ was reduced or interrupted in *ph* clones surrounded by wild-type tissue where *wg*^HA^ was normally expressed in regions with differentiating photoreceptor cells (Fig 6E and 6F). Even though *wg* can be secreted and potentially function at a longer range, as it does in wing disc patterning, our data suggest that this PcG target gene is also not globally upregulated in *ph* clones.

Finally, we analysed the expression of Distal-less, encoded by *Dll* and required for determination of ventral imaginal disc identity and development [39,40]. Dll expression is restricted to the antenna in wild-type eye-antennal discs, and with control discs with neutral clones (S5E and S5F Fig). Discs with *ph* clones also displayed a pattern restricted to the antenna and no staining was observed in clones growing in other disc locations (Fig 6G and 6G’). Furthermore, when clones grew in the antennal region the Dll staining was only found in tissue sections close to the surface (Fig 6H) but was interrupted at deeper z-sections where *ph* clones grew in the same disc (Fig 6I). Thus, the PcG target *Dll* also does not appear to be re-expressed in the absence of PRC1 silencing.

The previous examples challenge the notion of a full and generalized de-repression of PcG targets in *ph* clones. Instead, we considered that abrogation of PRC1 function could yield a permissive chromatin state but additional factors would be required to activate the expression of PcG targets. The relevance of *Cubitus interruptus* (*Ci*) reported years ago as a compartment-specific activator of *dpp* in *ph* clones [15], led us to consider that other transcription factors, namely downstream targets of signalling pathways, could have additional roles in activating Polycomb-silenced genes.

### The canonical JNK activator AP-1 is required for de-repression of a PRE in *ph* clones

Our previous results showed that simultaneous JNK and JAK/STAT signalling were not sufficient to induce tissue aberrations but loss of *ph* was also insufficient for broad de-repression of a selected number of PcG targets. We therefore hypothesized that a combination of activators and loss of Polycomb repression is required for context-specific target gene expression in *ph* tumorigenesis. Although it is difficult to predict the full extent of activators that could trigger genes previously inaccessible due to Polycomb silencing, we focused on effectors of relevant signalling pathways as potential candidates. For example, motifs for the canonical transcription factors downstream of JNK and JAK/STAT (AP-1 and Stat92E, respectively) have been associated with open chromatin regions in other tumours [41]. However, to test the possibility of a dual requirement of activators and loss of *ph* silencing, we sought to identify an element responsive to both inputs, ideally located close to a Polycomb response element (PRE) [42,43]. We recurred to a reporter of the pro-apoptotic gene *reaper* that became activated in response to loss of cell polarity in a JNK-dependent manner, and which we found to also harbour a PRE [34,43].

**Fig 7:**
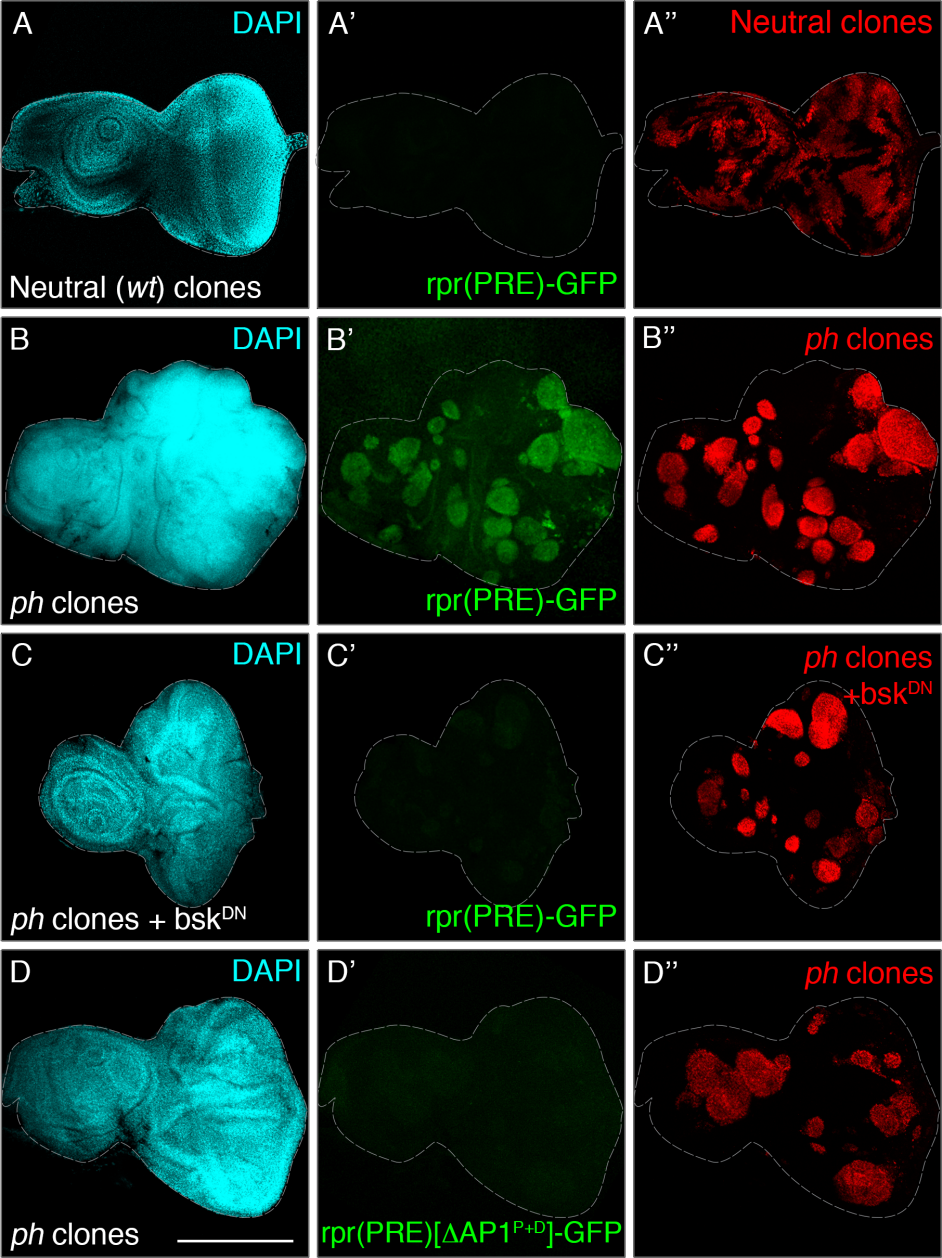
Signalling-dependent activators are required for de-repression of a PRE in *ph* mutant clones. **(A-A”)** The rpr(PRE)-GFP reporter is generally undetected in control eye discs with neutral clones. **(B-B”)** The reporter becomes activated in *ph* clones, but its expression is again reduced in clones expressing UAS-bsk^DN^ to block JNK signalling **(C-C”)**. **(D-D”)** Similar abrogation of reporter expression in *ph* clones was observed when using a version with mutated binding sites for the canonical JNK transcription factor AP-1. DAPI staining (cyan) is shown in the left panels, while the central panels show the reporters and the distribution of RFP-marked clones is shown on the right panels. Scale bar represents 200 μm.

To functionally test the involvement of JNK signalling to activate PcG-target genes, we assessed expression of the rpr(PRE)-GFP reporter in discs with neutral or *ph* clones. We first checked the normal expression pattern of the rpr(PRE)-GFP reporter in wild-type eye-antennal discs (Fig 7A–7A”). Reporter expression was previously shown to require JNK-dependent inputs, which is generally absent in eye discs, and the reporter was generally silent throughout the disc. Interestingly, the reporter became readily detected in most *ph* clones, suggesting that it becomes responsive in absence of PRC1 repression (Fig 7B–7B”).

Next, we examined whether blocking JNK, through expression of UAS-bsk^DN^ in *ph* clones, had an effect on the rpr(PRE)-GFP reporter. We observed reduced reporter expression in this case (Fig 7C–7C”), compared to its marked expression in *ph* clones alone (Fig 7B–7B”). These observations suggested that the reporter becomes active in the absence of *ph* (and not in the surrounding wild-type tissue) in a JNK-dependent fashion. We built up on the previous analysis of specific AP-1 binding sites present in the reporter that were required for its expression in polarity-deficient cells [34]. We thus used the version of the reporter with mutated AP-1 sites, rpr(PRE) [ΔAP1^P+D^]-GFP, and observed a reduced expression in comparison to *ph* clones alone, similarly to blocking JNK with bsk^DN^ (Fig 7D–7D”).

We sought to extend this type of analysis to additional cis-regulatory elements containing PREs, some of which have recently been shown to have a dual function, also behaving as enhancers [44]. We obtained and tested 4 of the PRE reporter lines showing also enhancer function in the embryo (namely, bx-PRE, Scr10Xba.1-PRE, P{C4-418bis}-PRE, and eve-PRE300. Of these, 3 displayed ectopic expression when combined with *ph*, in eye disc clones, in comparison with a low-level patterned expression in control discs (S6A–S6H Fig). The exception was the *eve* PRE, reported to act as an enhancer in the embryo [44], which we found unaltered in *ph* clones in the eye disc (S6G and S6H Fig). This could be consistent with the notion that a putative activator responsible for activation in the embryo might not be present in the eye disc.

We also surveyed a resource of developmental enhancer elements (‘Vienna Tiles’ collection [45]) for other available reporters that overlapped with previously-mapped PcG binding sites [43]. We identified several enhancer reporter lines that contained PREs and combined 4 more with the *ph* mutation. Since this collection was developed with a Gal4 read-out, the MARCM system would be incompatible so we generated *ph* clones using the ubi-RFP twin-spot system [46], thus *ph* clones are negatively marked in this case (absence of RFP signal). Although the Gal4 antibody had some background, we could observe stronger staining within *ph* clones (non-RFP tissue) for 3 out of 4 tested Vienna-Gal4 enhancer reporters (S6I–S6L Fig). These were reporters in proximity of *charlatan* (*chn*, where responsive clones were mostly detected in eye disc regions anterior to the morphogenetic furrow), *Abd-A* (responsive clones tended to be in more posterior regions) and *Abd-B* (more common in clones posterior to the furrow, although we noted a weaker signal in this case). The last VT-Gal4 reporter did not show upregulation in *ph* clones, as its expression was uniform/low across the entire disc (both RFP+/- cells) (S6L Fig). Together, these data suggest that some cis-regulatory elements may have a dual function as enhancer reporter lines show de-repression in the absence of *ph* silencing. The region-specific biases that were observed will need to be tested with better reporters, yet are consistent with the proposed function of additional activators in a tissue- or region-specific manner.

The use of a reporter responsive to both loss of *ph* and the JNK transcription factor AP-1 supports a role for JNK-dependent activation of at least some PcG target genes in the absence of PRC1 function. These data suggest that loss of *ph* may render a permissive chromatin state at targets that are normally silenced during development, but additional factors such as signalling activators may also play a role in tumorigenesis. This points out that further regulatory complexity needs to be considered in addition to loss of repressive complexes in order to understand tumour-driving gene expression programs.

## Discussion

When we consider the lifespan and complexity of multicellular organisms, the emergence of cancer is a rather rare event, yet with severe destructive consequences. This points to the existence of fail-safe mechanisms that ensure tissue homeostasis and are often efficient in protecting the organism, by mounting a response to damage or eliminating abnormal cells arising from stochastic or mutagenic events. Motivated by shared similarities between faithful regenerative responses and tumour onset, which activate common pathways, we sought to dissect context-specific properties.

Here, we have identified a tumour-specific signalling hierarchy in tissues with dysfunctional Polycomb silencing in the absence of *ph*. Our data suggested that the function of additional activators may be required for *ph* tumourigenesis in addition to aberrant signalling modules. Furthermore, this suggests a higher complexity in driving tumorigenic gene expression than previously anticipated.

### Ectopic JNK, JAK/STAT and Notch signalling contribute to *ph* tumour development

Abnormal signalling has been widely reported in a many cancer types across species [1,47,48]. It is clear that cancer cells can utilize signalling modules to obtain growth advantage and invade healthy tissues, but it is often difficult to tease apart whether this is a consequence of transformation or an intrinsic feature of tumour onset. *Drosophila* tumour models are useful to study early transformation mechanisms, as genetic tools permit to study tumours after only a few rounds of cell division from the initiating clone.

We used specific reporters for three signalling pathways that were ectopically activated in *ph* tumours, and functionally tested to which extent they contributed to tumour growth (Figs 1 and 2). JAK/STAT and Notch signalling were previously noted to be upregulated in *ph* clones, and we also observed JNK activation in the majority of clones [16–18]. We developed a quantitative framework to measure the functional effect of blocking the three relevant pathways and found that all contribute to tumour growth (Fig 2F, 2I and 2L).

Impairment of JNK and JAK/STAT yielded a reduction in clone volume, as shown by a shift of the volume taken by large clones to mostly small or very few medium clones, but the number of clones arising was not greatly altered (compare Fig 2F and 2I to 2C). Impairment of Notch also resulted in a marked decrease of clone volume, but in this case the number of recovered clones also decreased slightly (Fig 2L).

Applying similar approaches to other tumour models could be useful, as representative data will hardly translate the intrinsic complexity of tumour structures.

### Crosstalk between signalling pathways activated in *ph* tumours

JNK, JAK/STAT and Notch have been implicated in a wide variety of processes, from normal development to damage responses or even other tumours [1,8,11,18,19,49]. JNK emerges as a central stress pathway activated in several contexts and leading to a range of behaviours including apoptotic cell elimination [4,9,19,34]. It is commonly activated in tumours and is required for invasion and malignant phenotypes, mediated for instance by its target Mmp1 or in cooperation with other transcription factors [12,13,50,51]. JAK/STAT has been linked with stem cell niches [49] but is also expressed in developing discs ([23] and Fig 1E”) as well as during regeneration [19]. A recent report proposes that, in response to tissue damage, JAK/STAT signalling enables cells with active JNK to survive [36], and pro-survival cues have also been described during appendage development and specification [52]. Finally, Notch has been long known to induce tissue hyperplasia, and can promote tumorigenesis in cooperation with additional factors [9,11,53]. Following the observations that some of these pathways were activated in situations leading to opposing outcomes, such as regeneration and tumour formation, we dissected the signalling hierarchy characteristic of *ph* tumours.

Blocking JNK signalling did not greatly impact on JAK/STAT but led to significant abrogation of the Notch reporter (Fig 3). We were at first surprised by the lack of a dependency of JAK/STAT on JNK, as the ordered activation of the JAK/STAT ligand *upd* was shown to be JNK-dependent during disc regeneration [19]. However, de-repression of *upd* ligands was previously shown in *ph* tumours, which could render a deregulated state to allow parallel signalling [17]. JNK was also shown to positively regulate an enhancer of upd3 in wing discs with disrupted polarity by knocking down *dlg* [18]. However, since there are additional ligands for the pathway, there could be alternative means to activate it. These observations, together with the data presented here, indicate that alternative mechanisms likely activate the JAK/STAT pathway in *ph* tumours, since JAK/STAT activity could still be detected with the 10xSTAT reporter. We also considered that non-canonical signalling activities were common in tumours unlike in developmentally regulated contexts, pointing to possible differences that might underlie distinct outcomes. We thus asked if conversely blocking JAK/STAT had an effect on JNK, and found that these two modules do seem to act in parallel in *ph* tumours (Fig 4).

Furthermore, Notch signalling was also unaffected by blocking JAK/STAT (Fig 4), and impairing Notch signal transduction led to abrogation of its own reporter but had no effect on JNK and a mild effect on JAK/STAT (Fig 5). These data suggested that Notch could function downstream of JNK, but this axis likely acts in parallel to JAK/STAT (model in S7 Fig). The slight effect on the JAK/STAT reporter was mostly contributed by its decrease in smaller clones, which may be connected to modulation of survival in response to JNK, as shown to be mediated by JAK/STAT in tissue recovery [36]. It can also be due to Notch-mediated regulation of *upd*, which had previously been shown during eye disc development [28–30]. Whether cell-autonomous or non-autonomous regulation occurs in *ph* clones remains to be tested, as both cases were previously reported [28,29]. It will be interesting to examine in more detail whether Notch can play a similar role in promoting survival, which can be expected due to its ability to induce tissue hyperplasia and also by our observations that blocking Notch led to a decrease in the number of recovered *ph* clones (Fig 2L).

Despite the plethora of responses directed by all these pathways, delineating epistatic relationships among them has been done in a small range of contexts. An elegant exception was the identification of a synergistic cooperation between Src/JNK and activated Notch, leading to increased malignancy in addition to hyperplasia [11]. In this situation, JAK/STAT was reported to act downstream of JNK, which was in turn activated by synergistic Src/N activation. However, N^act^ was used as the initial trigger to carry out a screen for modifiers of the hyperplasia phenotype. Therefore, despite the different hierarchies found in this study, the early requirement for Notch was an intrinsic feature of that model. Together, these studies point to a prevailing involvement of these pathways in supporting tumour formation, although there may be context-specific mechanisms that need to be further explored.

### Absence of *ph* renders a permissive state that may require additional activators

Intrigued by the parallel function of JNK and JAK/STAT in *ph* tumours, in contrast to a rather linear sequence during regeneration [19], we asked whether collective signalling through both cascades could yield tumorigenic-like properties. However, expression of upstream components triggering JNK or JAK/STAT, either alone or in combination, did not seem to recapitulate aberrations typically seen in tumours (S4 Fig). Thus, we reasoned that additional elements were required to develop tumours in addition to simultaneous JNK and JAK/STAT. We thus asked how genes could become deregulated in tumourigenesis, in particular how PcG targets that are silenced during development get de-repressed in *ph* tumours. Although some examples were previously examined, namely members of the Notch and JAK/STAT pathways [16,17,54], we found that de-repression may be dependent on additional regulation. We showed that at least three PcG target genes were not generally expressed throughout the majority of *ph* clones, and their endogenous pattern was rather interrupted when *ph* clones grew in those regions of the disc (Fig 6). Thus, loss of *ph* seems to render a permissive state that may still require positive inputs for expression of PcG targets, rather than global de-repression. Together with early observations of irradiation-induced *ph* clones in wing discs, where upregulation of several PcG targets was compartment-specific [15], these data suggest a more complex modulation of gene expression in tumorigenesis. There could be a role for other activators, as proposed for *Ci*-dependent regulation of dpp-lacZ in *ph* clones solely in the anterior compartment of wing discs [15].

We propose that transcription factors acting downstream of the involved signalling pathways seem reasonable candidate activators to counteract reduced Polycomb repression, which will need to be broadly tested in future studies. We hypothesized that a combinatorial function of activators in a permissive state resulting from abrogation of PRC1 could synergistically contribute to tumorigenesis in *ph* clones. Interestingly, a recent study revealed that some PREs can have a dual functionality as enhancer elements [44]. Some enhancers and their target genes were also shown to become more broadly expressed in embryos lacking *ph*, favouring the possibility that developmental transcription factors can activate target genes in broader domains when Polycomb silencing is abrogated. This is also supported by our observations that 6/8 additional PRE reporters with enhancer functions became de-repressed in *ph* clones (S6 Fig), some of them with a region-specific pattern, resembling the previous report of *dpp* in wing discs [15].

We used a reporter that integrated JNK inputs and contained a PRE, enabling us to test the possibility of overlapping requirements in a particular context. In this case, we had a data-driven candidate regarding a particular activator, based on previously described JNK input [34]. We observed that reporter expression in *ph* clones required JNK-dependent inputs from the canonical transcription factor AP-1 (Fig 7), suggesting that both loss of repression and activators may play a role to drive tumorigenic gene expression. In light of the dual functionality of cis-regulatory elements as PREs and enhancers [44], the absence of *ph* may lead to a state where some ectopic activators become able to target enhancers that were previously unavailable. This would also be in line with a recent observation that embryonic H3K27me3 prevents unscheduled accumulation of H3K27ac (active enhancer mark) at regulatory regions, with precocious activation of lineage-specific genes [55]. The global effects in altered chromatin dynamics, and the involvement of signalling-dependent and other activators, remain to be elucidated.

Our analyses established a signalling hierarchy in *ph* tumours that does not follow the ordered activation of JAK/STAT downstream of JNK as shown in regeneration. These differences may be at the basis of distinct outcomes despite utilization of the same modules, as it is currently unknown how signalling inputs are integrated to achieve a variety of cellular behaviours. For instance, JNK has been implicated in transient downregulation of PcG silencing to allow cellular reprogramming during regeneration [56], but full abrogation of *ph* likely results in a peculiar chromatin state. Alterations in the local chromatin environment may permit signalling-induced transcription factors to target previously inaccessible elements. Thus, the overlap of signalling-dependent regulation with context-defining chromatin states may prime tissue responses towards faithful or damaging outcomes. It will be rewarding to explore how signalling pathways can instruct different responses at a genomic level, which will be aided by emerging technologies.

## Materials and methods

### *Drosophila* genetics and fly stocks

The MARCM/eyFlp system was used to generate clones in eye-antennal discs, following the previously established model for *ph* tumorigenesis [16]. Crosses and stocks were generally maintained at 25°C. The following stocks were used: yw, FRT19A (blank FRT used as control (BL1744)); ph^505^, FRT19A/FM7, act-GFP (FAG); tub-Gal80, FRT19A; eyFlp5, ac>y^+^>Gal4, UAS-GFP (‘19ATesterGFP’, T. Xu) [16]. Tester stocks with act>>RFP and act>>lacZ variants were also used to generate clones in combination with fluorescent reporters (tub-Gal80, eyFlp, FRT19A; act>CD2>UAS-RFP/TM6 (from BL30558) and ph^505^, FRT19A/FAG; act>stop>UAS-lacZ/TM6 (from Hugo Stocker)). The ph^505^ line was also combined with: TRE-DsRed (JNK reporter) [21]; 10xSTAT-GFP [23]; NRE-EGFP [24]; UAS-bsk^DN^ [25]; UAS-dome^ΔCYT^ [26]; UAS-N^DN^ [27]; UAS-egr(RNAi) (BL58993); UAS-grnd-RNAi (VDRC #GD43454); wg^HA^ [38]; dpp-lacZ (T. Katsuyama); rpr-5.5-GFP and rpr-5.5-GFP[ΔAP-1^P+D^] [34]. FRT19A was also combined with UAS-hep^act^ [33]; UAS-hop [35], as well as tested with UAS-bsk^DN^, UAS-dome^ΔCYT^, UAS-N^DN^, wg^HA^ and dpp-lacZ (as controls).

To test the role of PRE and enhancer elements, we obtained four PRE lacZ reporter lines generated with phiC31-mediated integration (from Eileen Furlong), which we combined with the *ph*^*505*^ line: bx-PRE-lacZ, ScrXba.1-PRE-lacZ, P{C4-418bis}-PRE-lacZ and evePRE300-lacZ [44]. We additionally tested 4 other developmental enhancer Gal4 reporters from the ‘Vienna Tiles’ collection [45], which contained regulatory regions of PcG binding [43]: VT017013 (closest gene: *chn*); VT42795 (nearest gene: *Abd-A*); VT042867 (assigned to *Abd-B*); and VT033934 (in the proximity of CG34260). Since the read-out was Gal4 in these cases (making it incompatible with MARCM clones), ph^505^ clones were generated with the ubi-RFP twin-spot system [46], crossing to Ubi-mRFP.nls, w, FRT19A; eyFlp/ SMTM (combined from BL# 31416 and BL#5579), and thus mutant clones are marked by the absence of RFP while neighbouring wild-type cells retain RFP fluorescence (see S6 Fig).

### Staining procedures and antibodies

Dissection of larval tissues was done in PBS, fixed in 4% paraformaldehyde/PBS for 20 minutes and washed with PBS, 0.1% Triton X-100. DAPI staining was routinely done for 10 minutes (with endogenous fluorescence or after staining with secondary antibodies), 1/1000. The appropriate secondary antibodies routinely used were Alexa-fluor 488, 555, 568 or 647 (Life Technologies). Wandering L3 larvae were routinely selected (against marked balancer chromosome) for dissection of eye-antennal discs; due to the developmental delay of *ph* tumour-bearing larvae, 6-day old larvae were dissected for this condition (crosses were kept at 25°C with a 18h-24h laying period with subsequent aging until dissection). Discs were mounted in Vectashield medium (Vector Laboratories). For immunostaining, blocking was done for 1 h at room temperature with PBS, 0.1% Triton, 0.1% Bovine Serum Albumin (BSA). The following antibodies were used: mouse anti-β-galactosidase (Promega, 1/400); mouse anti-Gal4 (Santa Cruz, 1/50); mouse anti-HA (Sigma, 1/400); guinea pig anti-Distal-less (from M. Affolter, University of Basel, 1/2000); mouse anti-Delta (DSHB C594.9B-a, 1/700); rat anti-Serrate (a kind gift of Ken Irvine, 1/1000) [57]; mouse anti-NICD (DSHB, C17.9C6, 1/300).

### Confocal microscopy and image analysis

Images of imaginal tissues were acquired using 20x (or 40x oil) objectives on the Leica SP5/SP8 confocal microscopes (maintained with help from Single Cell Facility, D-BSSE, ETHZ), and processed using Fiji/ImageJ or Photoshop. Fiji was also used to determine the number of total clones and those with reporter activity upon blocking different signalling pathways.

### Quantitative measurements of *ph* clone volumes

We developed a pipeline to obtain accurate measurements of GFP-marked clone volumes in eye discs. To automate image segmentation and identification of clones across imaginal discs, we used Ilastik (Interactive Learning and Segmentation Toolkit, ilastik.org) to build an unbiased supervised learning classification of clone regions and surrounding tissue (with 4–5 discs). Confocal images of tumoral discs were acquired with a 0.8–1 μm z-stacks, usually with 28–35 stacks per disc. The classification method was then used for the test set of discs (up to 50 per condition) as well as upon blocking each signalling pathway. After unbiased classification of clones, a Matlab script (kindly developed by Aaron Ponti, SCF, D-BSSE) was used to enable us to use Imaris (Bitplane) to obtain volume data for each spatially defined clone, and the respective total clone number per disc. This methodology is therefore useful to robustly obtain tri-dimensional (volume) information of tumour clones, which have a considerable z-component, while control clones remain in the epithelial plane and thus ‘clonal area’ is generally measured in the field. This approach enabled us to obtain a distribution of clone volumes grouped into three categories (small, medium and large), fitting their contribution to the total tumour (GFP) volume per disc (large: more than 20%, medium: 2–20%, small: less than 2%). We could then build upon this quantitative description when blocking each signalling pathway, and thus capture the full dynamic range of the response in an unbiased manner.

For control (neutral FRT19A clones) in otherwise wild-type discs (or with transgenes to block signalling, see S2C–S2G Fig), we measured the area occupied by the clones (GFP) and the remaining eye-antenna disc tissue, since these discs were flat (clone volume would not be appropriate for these). We used z-projection of ~3–6 stacks per disc, to obtain the focus of the epithelial layer and thus measure the maximum area of the flat disc. We then segmented the GFP-marked regions (with an automated tool to avoid detection by the user) within the DAPI-delimited tissue outline and measured the area taken by GFP and non-GFP territories, both in ‘black FRT19A’ clones only, and those additionally expressing transgenes to block the three pathways studied. We plotted the proportion of the GFP/non-GFP regions measured in each condition (see S2C–S2G Fig). Plots and statistics were generated with the Prism 7 software.

## Supporting information

S1 Fig The *Drosophila* TNF receptor *grindelwald* and its ligand *eiger* mediate JNK activation in *ph* tumours. **(A-D)** Matrix metalloprotease-1 (Mmp1), a downstream target of JNK signalling in several tumour models, is upregulated in eye-antennal discs bearing *ph* clones **(A)**, which is suppressed upon additional expression of bsk^DN^ **(B)** or knockdown of *egr* **(C)** or *grnd* **(D)**. Left panels display tissue morphology (DAPI staining); *ph* clones (or with additional genetic manipulations as indicated in each panel) are shown in the second column (in green) and Mmp1 staining on the third column (magenta); the last column depicts merged signals from the previous two. **(E-F)** Knockdown of either *egr* **(E)** or its receptor *grnd* **(F)** in *ph* clones leads to a reduction in clone size, also seen in (C,D), and prevents the ectopic expression of the JNK reporter TRE-DsRed (shown in the third column in this case as labelled) (compare with Fig 1A”). Further quantification of the effects in clone size detailed in S2 Fig. Scale bar represents 200 μm.
(TIF)

S2 Fig Detailed quantification of genetic manipulations on tumour growth and control tissues. **(A)** Additional representation of the clone volume data shown in Fig 2, here depicting data for whole tissue volume (DAPI) and total tumour volume (GFP) measured across 50 discs with *ph* clones (bars represent average and error bars represent standard error). This enabled to determine the distribution of the average proportion of tumour volume (total GFP volume per disc divided by total disc DAPI volume), shown in **(B)**. The proportion of tumour volume in each of the three categories (small, medium, large) is further detailed in **(B’)**, showing that the relative contribution from each ‘size’ category remains generally consistent. Of note, most discs had only 1–3 ‘big’ clones, which contributed to a significant proportion of the total tumour volume per disc (from 22% to 60%). The number of clones ranged on average between 11–20 for ‘medium’ and 13–22 for ‘small’. **(C-G)** As controls, clone size was measured for discs with neutral clones or additionally expressing the dominant negative constructs used to block the three signalling pathways. The discs showed an overall similar morphology as well as the clones (examples shown in **(D-G)**, as labelled in each panel). As clones respected the epithelial layer, area was measured in this case for all conditions. The relative area taken by GFP+ cells and non-GFP tissue area, per disc, is shown in **(C)**, and no significant differences were detected across these conditions (paired t-test comparing to neutral clones only). The number of discs analysed per condition was n = 29 (control discs with neutral clones), n = 20 (neutral clones with bsk^DN^), n = 23 (dome^ΔCYT^), and n = 25 (N^DN^). **(H,I)** Representative discs with *ph* clones with knockdown of *egr* (H) and *grnd* (I), and quantified in **(J)** and **(K)**, respectively (n = 23 discs for *egr*, n = 21 for *grnd*). The scale of the plots showing clone volumes for each of the three volume categories in **(J,K)** was maintained to enable direct comparison with Fig 2C. **(L)** Average tumour volume per disc across all conditions analysed, to simplify comparison between all genetic backgrounds and grouping the components tested for each of the three pathways. Scale bar represents 200 μm.
(TIF)

S3 Fig The link between JNK and Notch signalling is ligand-independent. **(A-C)** The Notch ligand Delta (Dl) is endogenously expressed in a typical pattern, mostly in the photoreceptor region in eye imaginal discs (control disc with neutral clones shown in **(A)**). In each pair of panels, DAPI and GFP-marked clones are shown on the left and staining for the indicated component is shown on the right throughout the figure. **(B)** Despite severe disruption of tissue morphology in discs with *ph* clones, Dl staining is not upregulated in *ph* tumours and remains restricted to the typical pattern, with no additional effect detected when blocking JNK signalling **(C)**. **(D-F)** Another Notch ligand, Serrate (Ser), is upregulated in *ph* tumours **(E)** (compare to endogenous pattern in control discs in **(D)**), but remains upregulated upon blocking JNK signalling in *ph* clones despite the smaller clone size **(F)**. **(G-J)** Using an antibody against the Notch intra-cellular domain (NICD), discs with *ph* clones showed it is upregulated in tumours **(H)**; however, upon blocking JNK signalling **(I)**, the NICD expression pattern was more comparable to that of control discs with neutral clones **(G)**, where it is generally detected along the morphogenetic furrow, although we noted that this is not fully penetrant (some discs still showed higher NICD in some clones, hence with some variability as shown in **(H)**). Scale bar represents 200 μm.
(TIF)

S4 Fig Co-activation of JNK and JAK/STAT is not sufficient for tumour formation in the presence of functional PcG silencing. **(A-C)** Neutral clones induced with the MARCM system are shown to depict the random generation of GFP-marked clones (B) in control discs. DAPI staining in the left panels reflects tissue morphology. The merged channels are shown in C. **(D-K)** Expression of the specific reporters was assessed as positive control for activation of JNK **(D-G)** or JAK/STAT **(H-K)**. GFP-marked clones (E) were used to identify neutral clones expressing UAS-hep^act^, and expression of the TRE-DsRed reporter (F) was detected in or around these. The merged channels are shown in (G). RFP-marked clones (I) expressing UAS-hop are widespread throughout the disc, and the 10xSTAT reporter (J) is broadly expressed in the clones, but also in the endogenous pattern where it is observed in the antennal region and some photoreceptors in wild type discs, as shown in the merged panel (K). **(L-N)** Discs with neutral clones that simultaneously trigger JNK and JAK/STAT were generated by co-expression of both UAS-hep^act^ and UAS-hop (M), but no apparent tissue aberrations were observed in these conditions. Scale bar represents 200 μm.
(TIF)

S5 Fig Baseline expression patterns of PcG targets in control discs. **(A-F)** Expression of three known PcG targets in wild-type eye discs (left) and control discs with neutral clones (right), as a baseline reference for comparison with discs carrying *ph* clones (see Fig 6). Expression patterns (in magenta) were generally indistinguishable in wild-type discs and discs with neutral clones, as shown for **(A-B)** dpp-lacZ, **(C-D)** wg^HA^ and **(E-F)** Distal-less, Dll. Clones are marked in green and DAPI in cyan.
(TIF)

S6 Fig Probing the dual functionality of cis-regulatory elements as PREs and enhancers in *ph* clones. **(A-H)** PRE-lacZ reporters tested in eye discs that have been recently reported as developmental enhancers in the embryo [44]. β-galactosidase staining was detected in *ph* clones for three PREs, namely *bx*-PRE **(A)**, Scr10Xba.1-PRE **(C)**, P{C4-418bis}-PRE **(D)** (albeit slightly weaker than the previous). The endogenous expression of the reporters in control discs is shown on the right panels **(B,D,F)**, respectively. Although the eve-PRE300 was reported to be de-repressed in the embryo [44], the expression pattern in eye-antennal discs with *ph* clones **(G)** was similar to that of control discs **(H)**, pointing to an embryonic specificity for the activity of this enhancer/PRE. **(I-L)** Four additional developmental enhancers that overlapped with PcG binding sites were also tested (from the Vienna Tiles Gal4 collection): VT17013-gal4 (*chn*) **(I-I”)**, VT42795-gal4 (*Abd-A*) **(J-J”)**, VT42867-gal4 (*Abd-B*) **(K-K”)** and VT33934-gal4 (*CG34260*) **(L-L”)**. Tissue morphology is shown on the first row of this set (DAPI staining), and *ph* clones are now marked by the absence of RFP signal (red, second row) (twin-spot clones as the gal4 reporters would not be compatible with MARCM clones). Anti-Gal4 staining was used to detect reporter expression (bottom row, in green). Despite a more variable expression of these reporters in comparison to the classical PREs (A-H), ectopic reporter expression was detected in some *ph* clones, as highlighted with the dotted regions in magnified discs (**I”**,**J”**) and arrow-heads in **K”**. In these cases, not all *ph* clones showed similar reporter levels, there was a bias for de-repression in certain disc regions, e.g. anterior to the morphogenetic furrow in two cases (*chn*, *Abd-B*) or posterior to it (*Abd-A*). The last reporter tested **(L”)** was not responsive throughout the disc, showing a homogeneous background that similar to that seen in control discs. These reporters were selected from a collection of developmental enhancers and their function as PREs could thus be tested for the first three cases. Scale bars represent 200 μm.
(TIF)

S7 Fig Diagram depicting signalling hierarchies in *ph* tumours and regenerative tissue recovery. The current model underlying the crosstalk between signalling pathways in the context of *ph* tumorigenesis is represented on the left, while the involvement of the same modules during regeneration is shown on the right. Different signalling hierarchies are observed in each context and can thus point to distinct outcomes.
(TIF)
