# Supplementary figures and images for: Signalling crosstalk during early tumorigenesis in the absence of Polycomb silencing
Source: PLoS Genet. 2018 Jan 22;14(1):e1007187. doi: 10.1371/journal.pgen.1007187 (PMC5794193; doi:10.1371/journal.pgen.1007187)

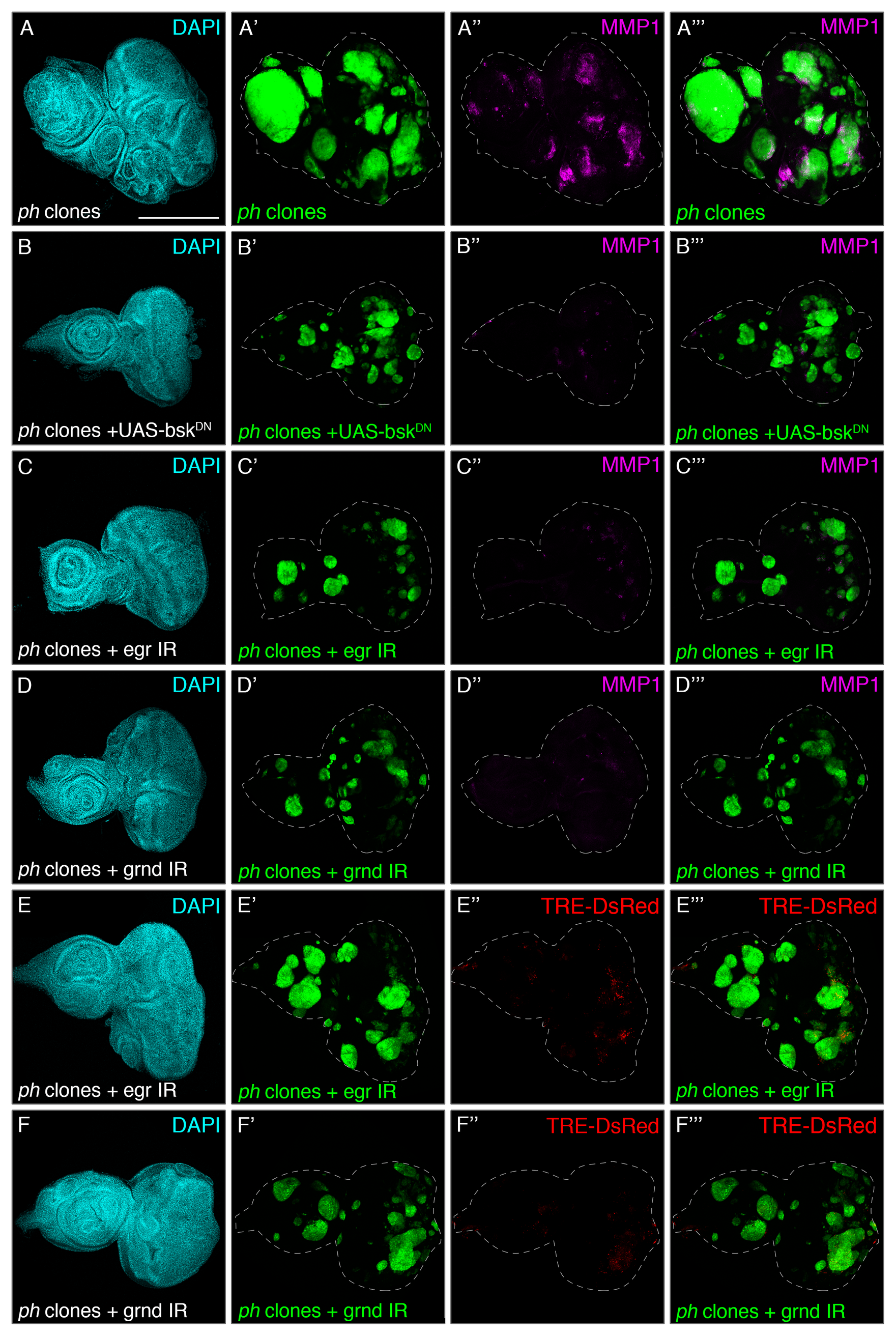

Supplement: S1 Fig — (A-D) Matrix metalloprotease-1 (Mmp1), a downstream target of JNK signalling in several tumour models, is upregulated in eye-antennal discs bearing ph clones (A), which is suppressed upon additional expression of bskDN (B) or knockdown of egr (C) or grnd (D). Left panels display tissue morphology (DAPI staining); ph clones (or with additional genetic manipulations as indicated in each panel) are shown in the second column (in green) and Mmp1 staining on the third column (magenta); the last column depicts merged signals from the previous two. (E-F) Knockdown of either egr (E) or its receptor grnd (F) in ph clones leads to a reduction in clone size, also seen in (C,D), and prevents the ectopic expression of the JNK reporter TRE-DsRed (shown in the third column in this case as labelled) (compare with Fig 1A”). Further quantification of the effects in clone size detailed in S2 Fig. Scale bar represents 200 μm. (TIF) [file pgen.1007187.s001.tif]

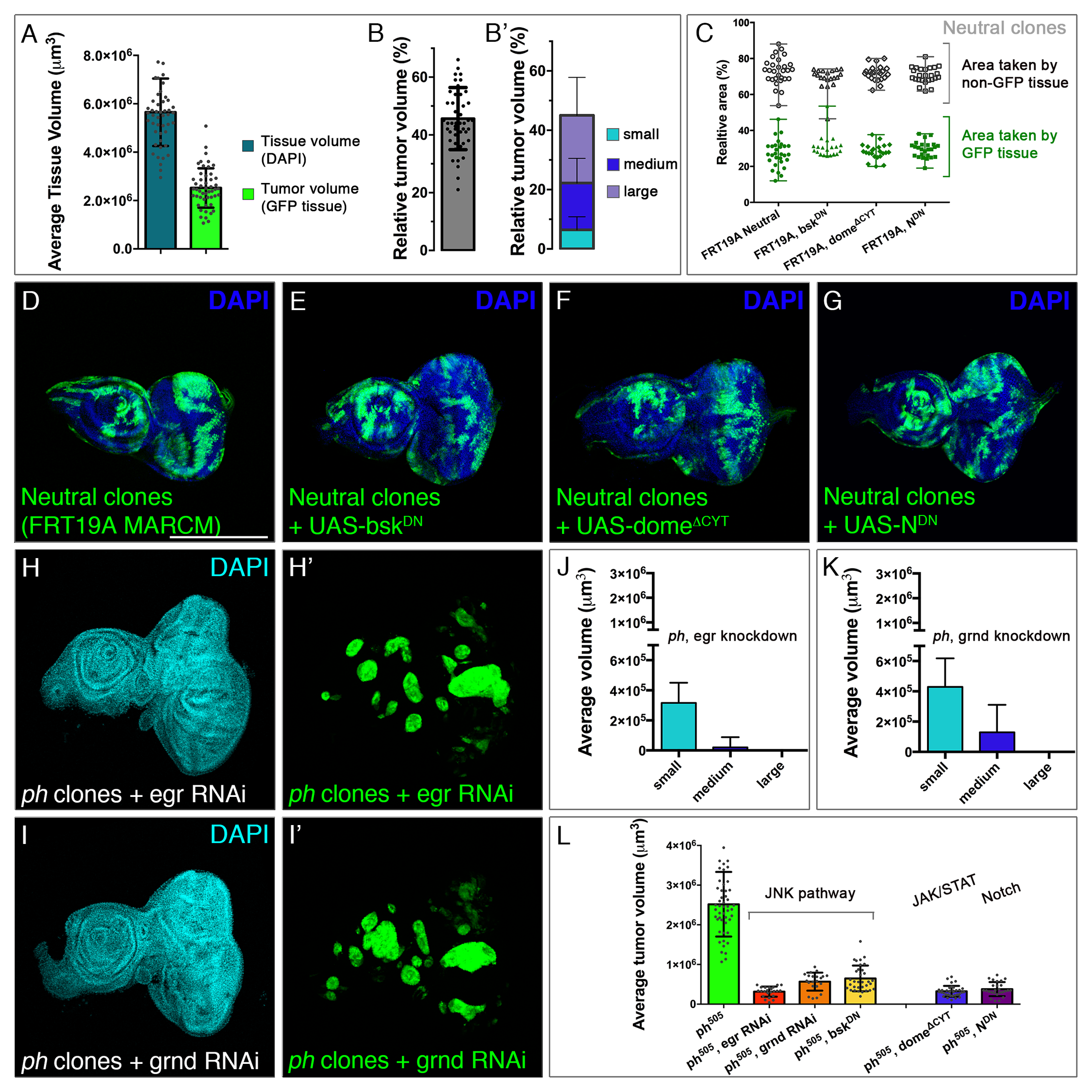

Supplement: S2 Fig — (A) Additional representation of the clone volume data shown in Fig 2, here depicting data for whole tissue volume (DAPI) and total tumour volume (GFP) measured across 50 discs with ph clones (bars represent average and error bars represent standard error). This enabled to determine the distribution of the average proportion of tumour volume (total GFP volume per disc divided by total disc DAPI volume), shown in (B). The proportion of tumour volume in each of the three categories (small, medium, large) is further detailed in (B’), showing that the relative contribution from each ‘size’ category remains generally consistent. Of note, most discs had only 1–3 ‘big’ clones, which contributed to a significant proportion of the total tumour volume per disc (from 22% to 60%). The number of clones ranged on average between 11–20 for ‘medium’ and 13–22 for ‘small’. (C-G) As controls, clone size was measured for discs with neutral clones or additionally expressing the dominant negative constructs used to block the three signalling pathways. The discs showed an overall similar morphology as well as the clones (examples shown in (D-G), as labelled in each panel). As clones respected the epithelial layer, area was measured in this case for all conditions. The relative area taken by GFP+ cells and non-GFP tissue area, per disc, is shown in (C), and no significant differences were detected across these conditions (paired t-test comparing to neutral clones only). The number of discs analysed per condition was n = 29 (control discs with neutral clones), n = 20 (neutral clones with bskDN), n = 23 (domeΔCYT), and n = 25 (NDN). (H,I) Representative discs with ph clones with knockdown of egr (H) and grnd (I), and quantified in (J) and (K), respectively (n = 23 discs for egr, n = 21 for grnd). The scale of the plots showing clone volumes for each of the three volume categories in (J,K) was maintained to enable direct comparison with Fig 2C. (L) Average tumour volume per disc across all c [file pgen.1007187.s002.tif]

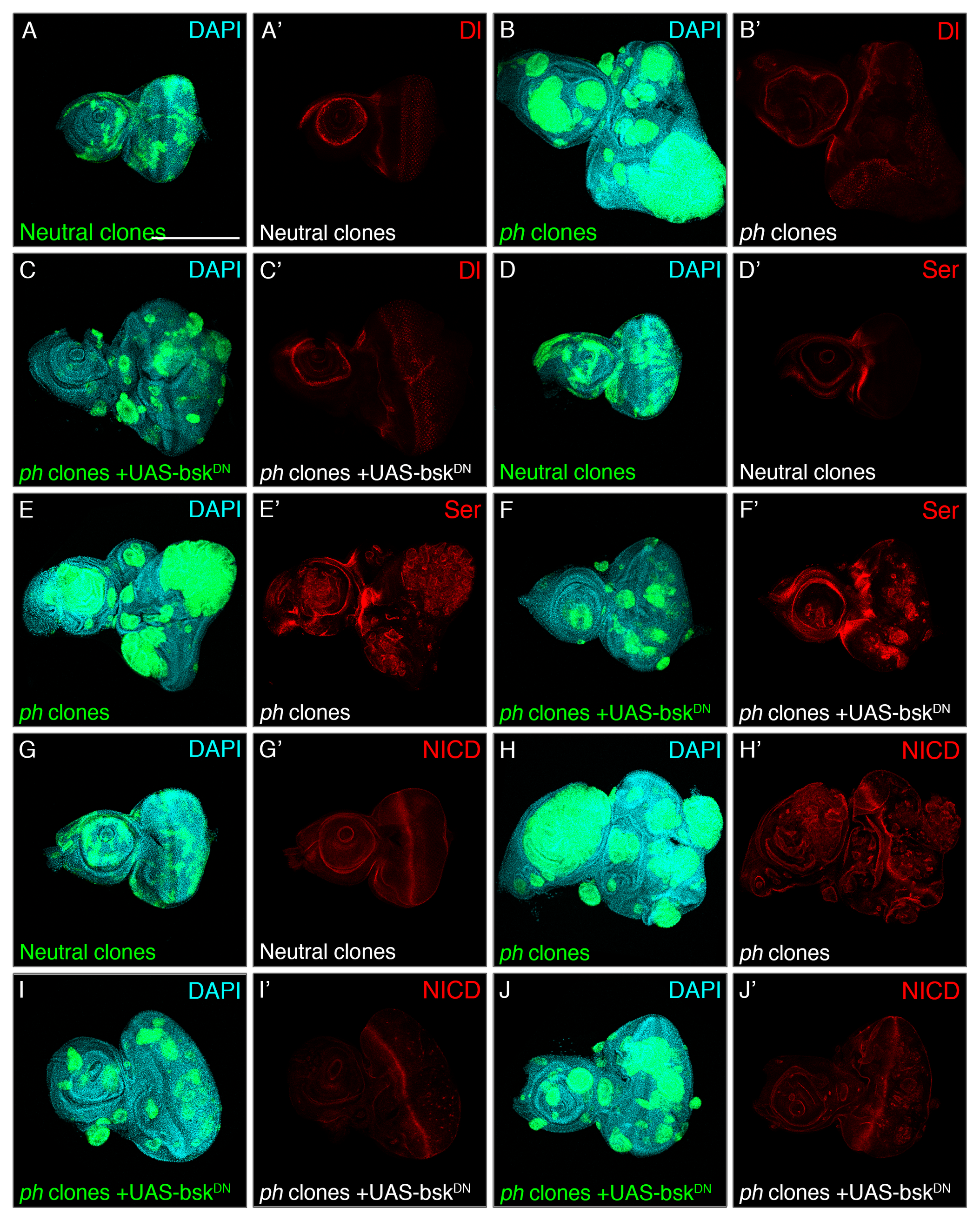

Supplement: S3 Fig — (A-C) The Notch ligand Delta (Dl) is endogenously expressed in a typical pattern, mostly in the photoreceptor region in eye imaginal discs (control disc with neutral clones shown in (A)). In each pair of panels, DAPI and GFP-marked clones are shown on the left and staining for the indicated component is shown on the right throughout the figure. (B) Despite severe disruption of tissue morphology in discs with ph clones, Dl staining is not upregulated in ph tumours and remains restricted to the typical pattern, with no additional effect detected when blocking JNK signalling (C). (D-F) Another Notch ligand, Serrate (Ser), is upregulated in ph tumours (E) (compare to endogenous pattern in control discs in (D)), but remains upregulated upon blocking JNK signalling in ph clones despite the smaller clone size (F). (G-J) Using an antibody against the Notch intra-cellular domain (NICD), discs with ph clones showed it is upregulated in tumours (H); however, upon blocking JNK signalling (I), the NICD expression pattern was more comparable to that of control discs with neutral clones (G), where it is generally detected along the morphogenetic furrow, although we noted that this is not fully penetrant (some discs still showed higher NICD in some clones, hence with some variability as shown in (H)). Scale bar represents 200 μm. (TIF) [file pgen.1007187.s003.tif]

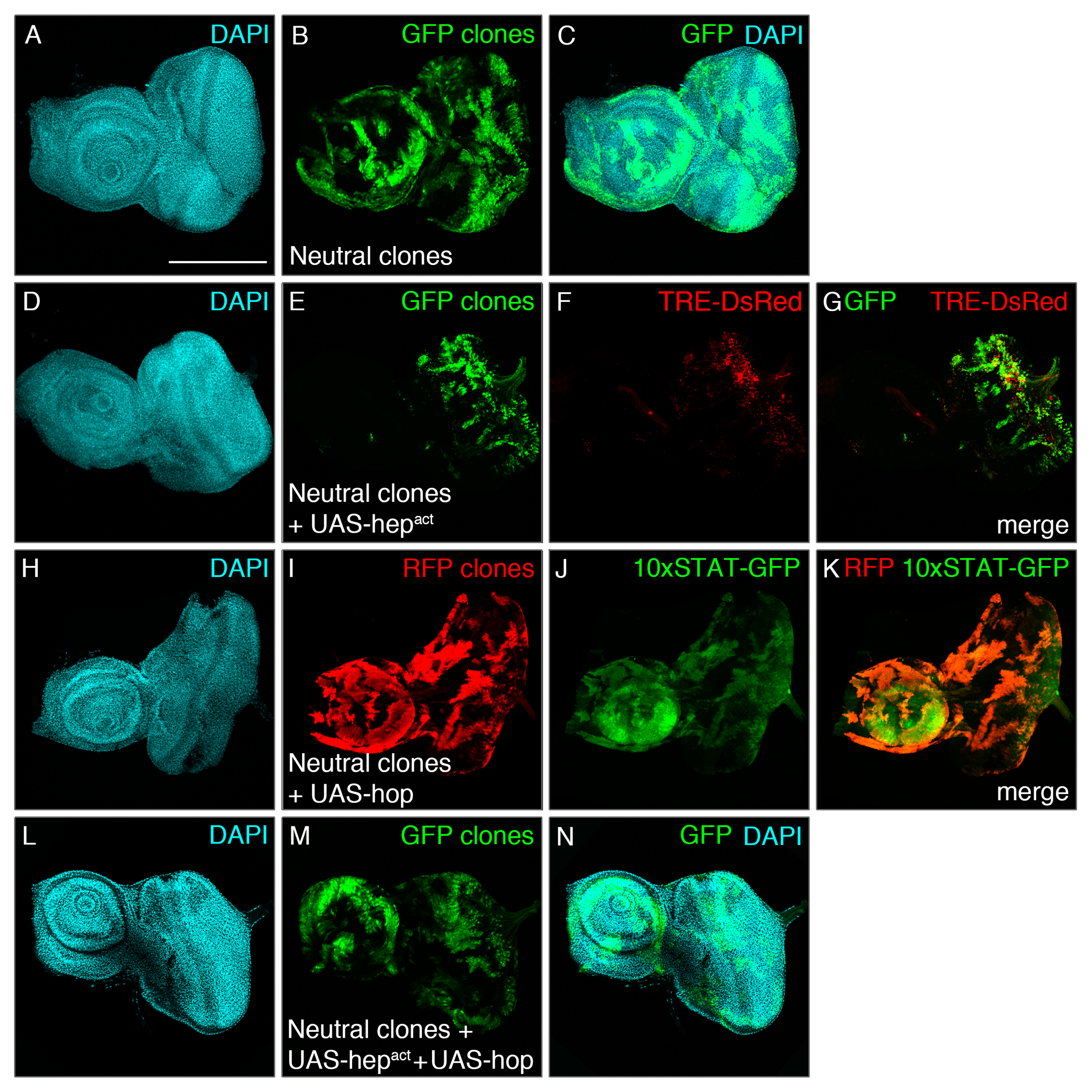

Supplement: S4 Fig — (A-C) Neutral clones induced with the MARCM system are shown to depict the random generation of GFP-marked clones (B) in control discs. DAPI staining in the left panels reflects tissue morphology. The merged channels are shown in C. (D-K) Expression of the specific reporters was assessed as positive control for activation of JNK (D-G) or JAK/STAT (H-K). GFP-marked clones (E) were used to identify neutral clones expressing UAS-hepact, and expression of the TRE-DsRed reporter (F) was detected in or around these. The merged channels are shown in (G). RFP-marked clones (I) expressing UAS-hop are widespread throughout the disc, and the 10xSTAT reporter (J) is broadly expressed in the clones, but also in the endogenous pattern where it is observed in the antennal region and some photoreceptors in wild type discs, as shown in the merged panel (K). (L-N) Discs with neutral clones that simultaneously trigger JNK and JAK/STAT were generated by co-expression of both UAS-hepact and UAS-hop (M), but no apparent tissue aberrations were observed in these conditions. Scale bar represents 200 μm. (TIF) [file pgen.1007187.s004.tif]

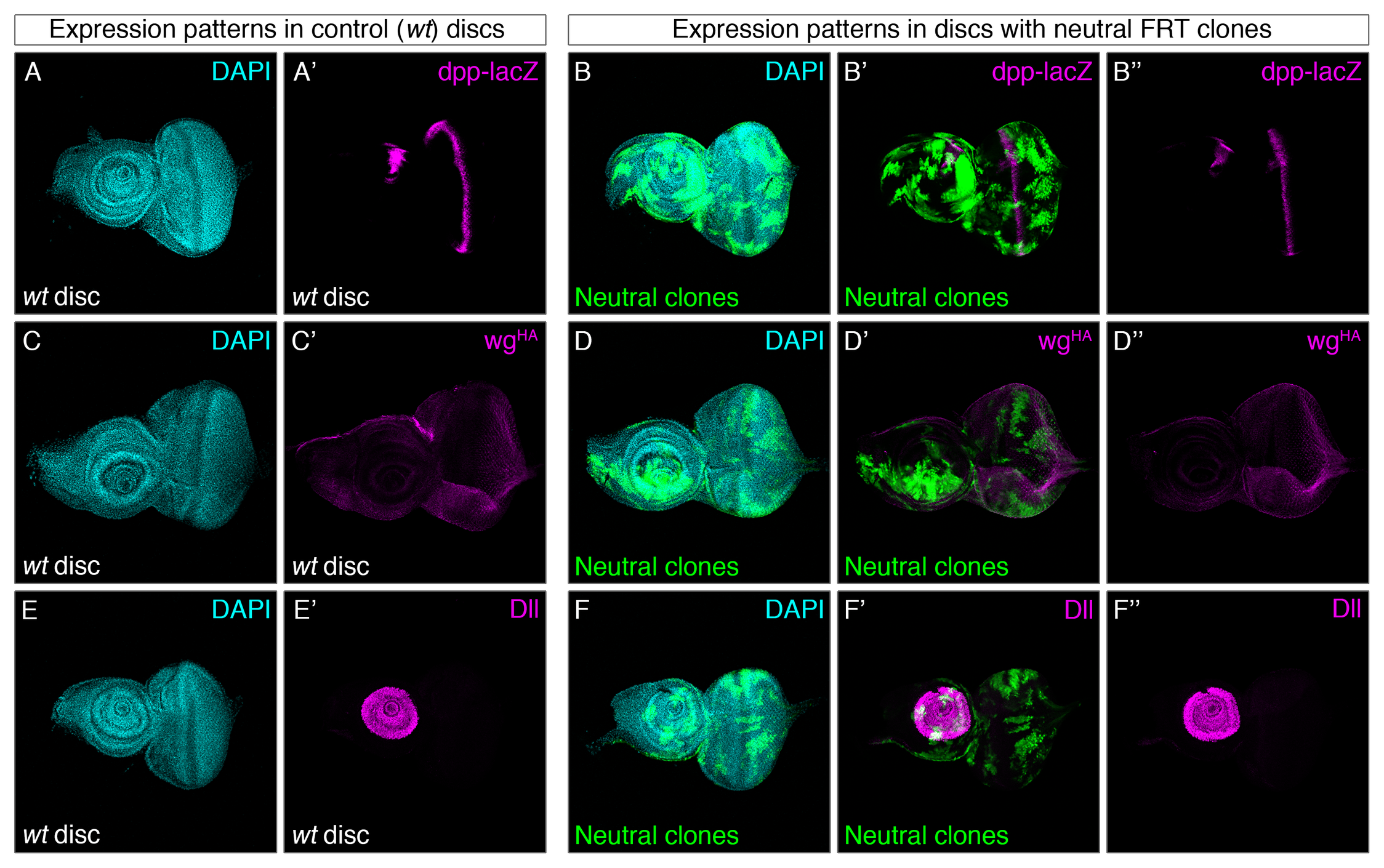

Supplement: S5 Fig — (A-F) Expression of three known PcG targets in wild-type eye discs (left) and control discs with neutral clones (right), as a baseline reference for comparison with discs carrying ph clones (see Fig 6). Expression patterns (in magenta) were generally indistinguishable in wild-type discs and discs with neutral clones, as shown for (A-B) dpp-lacZ, (C-D) wgHA and (E-F) Distal-less, Dll. Clones are marked in green and DAPI in cyan. (TIF) [file pgen.1007187.s005.tif]

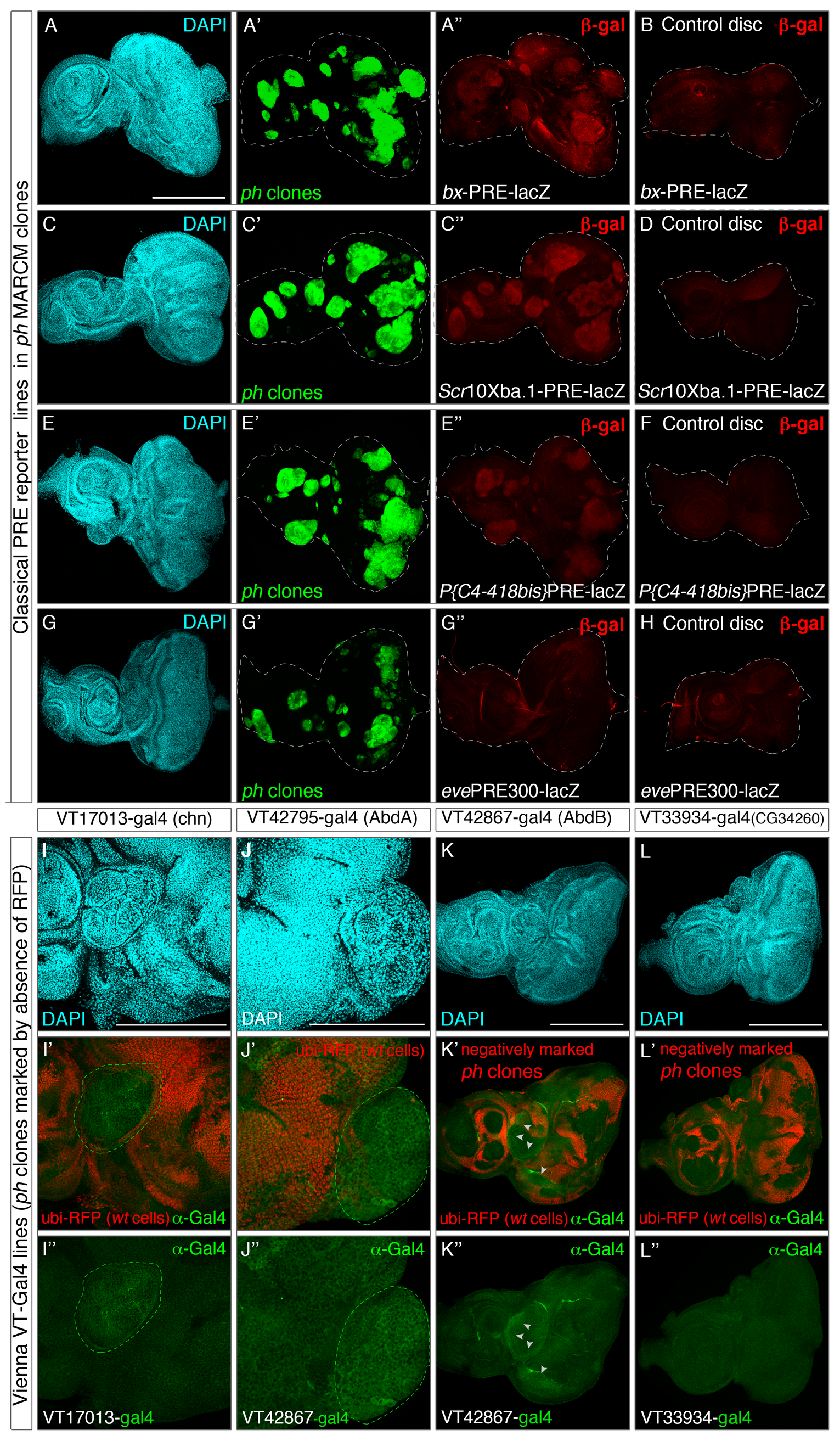

Supplement: S6 Fig — (A-H) PRE-lacZ reporters tested in eye discs that have been recently reported as developmental enhancers in the embryo [44]. β-galactosidase staining was detected in ph clones for three PREs, namely bx-PRE (A), Scr10Xba.1-PRE (C), P{C4-418bis}-PRE (D) (albeit slightly weaker than the previous). The endogenous expression of the reporters in control discs is shown on the right panels (B,D,F), respectively. Although the eve-PRE300 was reported to be de-repressed in the embryo [44], the expression pattern in eye-antennal discs with ph clones (G) was similar to that of control discs (H), pointing to an embryonic specificity for the activity of this enhancer/PRE. (I-L) Four additional developmental enhancers that overlapped with PcG binding sites were also tested (from the Vienna Tiles Gal4 collection): VT17013-gal4 (chn) (I-I”), VT42795-gal4 (Abd-A) (J-J”), VT42867-gal4 (Abd-B) (K-K”) and VT33934-gal4 (CG34260) (L-L”). Tissue morphology is shown on the first row of this set (DAPI staining), and ph clones are now marked by the absence of RFP signal (red, second row) (twin-spot clones as the gal4 reporters would not be compatible with MARCM clones). Anti-Gal4 staining was used to detect reporter expression (bottom row, in green). Despite a more variable expression of these reporters in comparison to the classical PREs (A-H), ectopic reporter expression was detected in some ph clones, as highlighted with the dotted regions in magnified discs (I”,J”) and arrow-heads in K”. In these cases, not all ph clones showed similar reporter levels, there was a bias for de-repression in certain disc regions, e.g. anterior to the morphogenetic furrow in two cases (chn, Abd-B) or posterior to it (Abd-A). The last reporter tested (L”) was not responsive throughout the disc, showing a homogeneous background that similar to that seen in control discs. These reporters were selected from a collection of developmental enhancers and their function as PREs could thus be tested for the first three [file pgen.1007187.s006.tif]

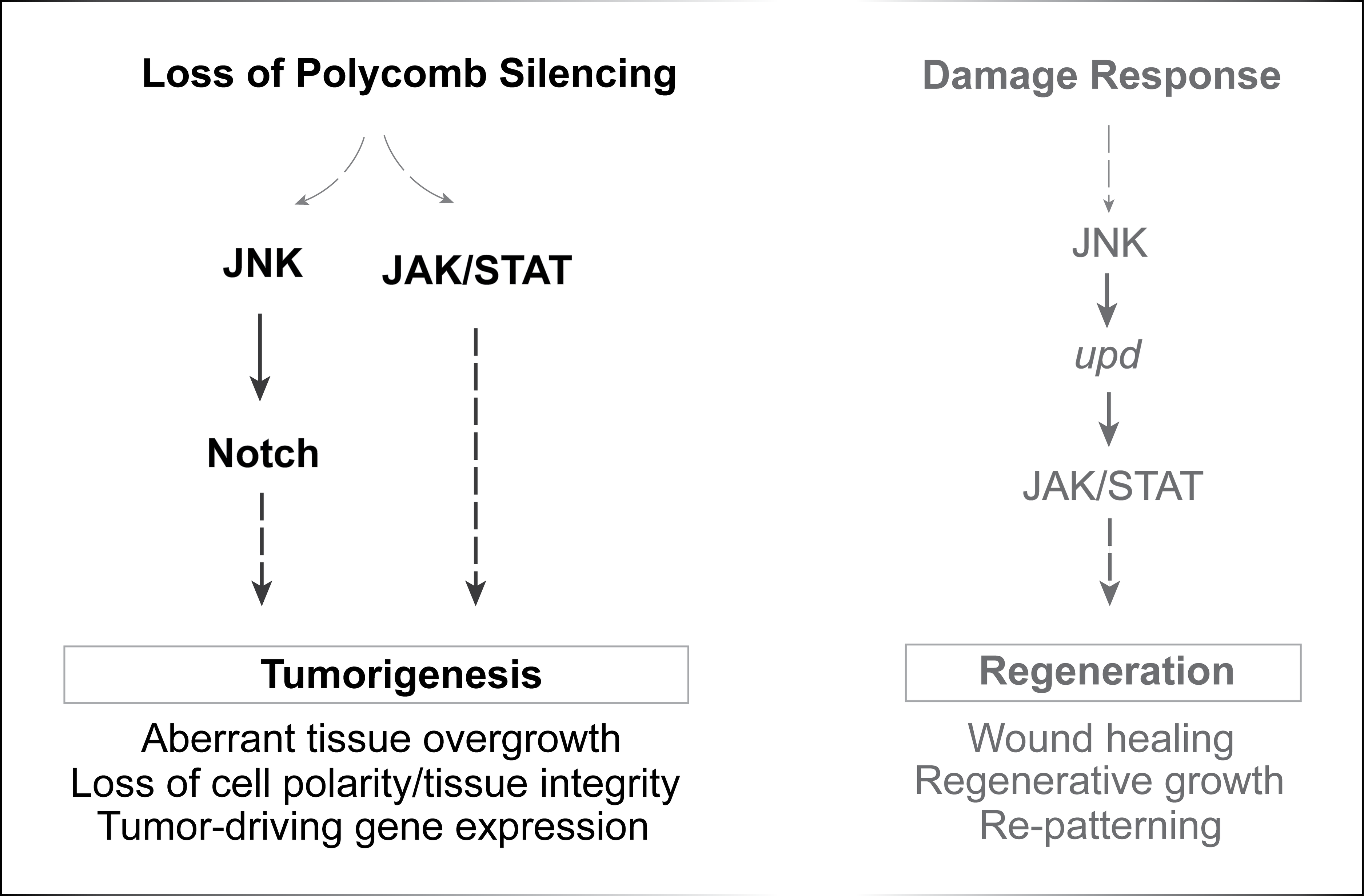

Supplement: S7 Fig — The current model underlying the crosstalk between signalling pathways in the context of ph tumorigenesis is represented on the left, while the involvement of the same modules during regeneration is shown on the right. Different signalling hierarchies are observed in each context and can thus point to distinct outcomes. (TIF) [file pgen.1007187.s007.tif]
